# Biobased Polymers in Printed Electronics: From Renewable Resources to Functional Devices

**DOI:** 10.3390/polym18020301

**Published:** 2026-01-22

**Authors:** Dimitra Karavasili, Kyriaki Lazaridou, Maria Angeliki Ntrivala, Andreas Chrysovalantis Pitsavas, Zafeiria Baziakou, Maria Papadimitriou, Nikolaos D. Bikiaris, Evangelia Balla, Ζoi Terzopoulou

**Affiliations:** 1 Laboratory of Polymer Chemistry and Technology, Department of Chemistry, Aristotle University of Thessaloniki, 54124 Thessaloniki, Greece; 2 Laboratory of Industrial Chemistry, Department of Chemistry, University of Ioannina, 45110 Ioannina, Greece

**Keywords:** sustainability, PE, flexible electronics, biodegradable materials, biosensors

## Abstract

Printed electronics (PE) have emerged as a rapidly growing technology owing to their potential for low-cost fabrication, flexibility, and scalable device manufacturing. The dependence on fossil-based components raises environmental concerns, leading the scientific community toward sustainable solutions, aiming to reduce the accumulation of electronic waste (e-waste) in the environment and the emission of toxic gases, as well as to offer a circular solution in the sector. This review presents an in-depth overview of biobased polymeric materials in printed and organic (bio-)electronics. Firstly, the principal printing techniques are presented in detail. The review proceeds by outlining the various biobased synthetic and natural polymers, along with their blends, that are employed in the fabrication of biobased substrates for printed devices. Finally, the review emphasizes the existing challenges and constraints in the field of PE, along with the promising opportunities for its future advancement.

## 1. Introduction

**Figure 1:**
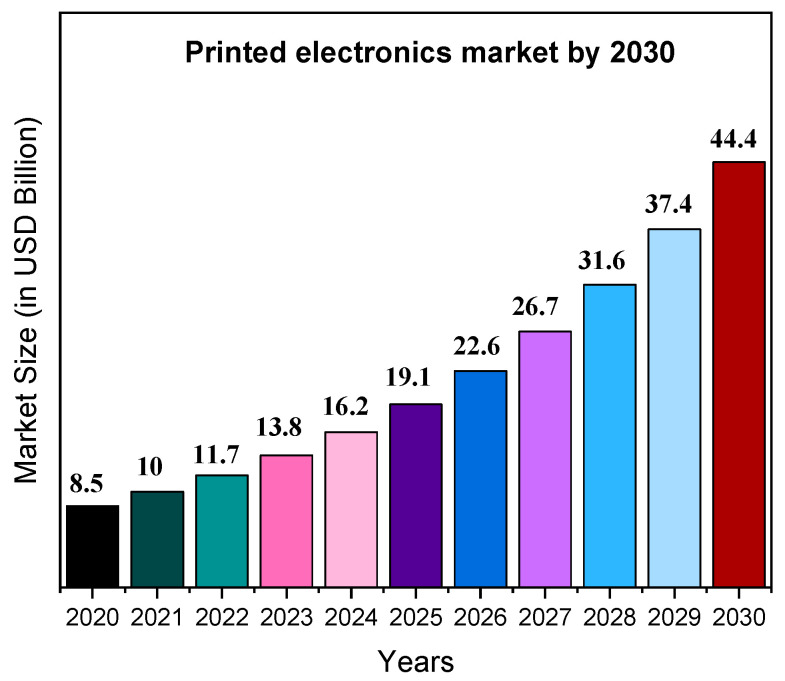
PE market by 2030 [5].

Printed electronics (PE) are one of the most rapidly developing additive fabrication technologies and are becoming invaluable for numerous applications [1]. This broad technical term refers to technologies used to manufacture electronic devices by printing on a wide range of substrate materials [2]. Overall, it encompasses processes for the fabrication of electrical circuits, components, and devices using traditional printing techniques. In 2020, it was estimated that 30 billion devices were connected to the Internet, encompassing a wide range of applications from smart cities to smart wearables, and extending from domestic environments to industrial settings. This expansion has been fueled by numerous advancements in electronics, polymer processing, and printing technologies [3]. Currently, PE are transitioning into their commercial phase, compelling academic research teams to address new challenges and issues that have only recently emerged [4]. It is indicative that the number of scientific studies on PE has increased since 2012, from nearly 600 articles to almost 1400 in 2021. Moreover, the PE market was estimated at USD 10 billion in 2021 and is predicted to reach USD 44.4 billion by 2030 (Figure 1), exhibiting a compound annual growth rate (CAGR) of 18.5% from 2021 to 2030 [5].

This rapid expansion in PE production is expected to lead to a substantial rise in the use of fossil-based materials that are currently prevalent in many components of PE, further intensifying environmental concerns due to their non-renewable nature and associated resource depletion [6]. To deal with such issues, researchers are making huge efforts toward the manufacture and use of biobased polymers, derived from natural and renewable sources, such as animals, plants, and microorganisms [7,8,9].

Biobased polymers, such as polyhydroxyalkanoates (PHAs), including poly(3-hydroxybutyrate) (PHB), cellulose acetate (CA), poly(lactic acid) (PLA), cellulose, and chitosan, have been exploited in the manufacture of flexible electronic devices, as printing substrates, dielectric or conductive layers, and active materials in sensors or biosensors [10]. Their adjustable macrostructure and high compatibility with other organic or inorganic materials make them an excellent basis for heterogeneous active materials in energy conversion and storage systems [11,12]. These polymers also exhibit notable stability, including thermal resilience up to 200–300 °C for PLA and PHB, mechanical durability under repeated bending or stretching, and resistance to environmental factors like humidity and mild solvents, enabling reliable long-term performance in flexible devices [13]. Despite their high stability, biobased polymers often receive targeted modification strategies in demanding flexible electronics applications to further enhance their overall robustness and performance. Common approaches include blending with nanofillers to improve mechanical strength, gas barrier properties, and moisture resistance, chemical cross-linking or plasticization to optimize flexibility and thermal endurance while preserving biodegradability, and surface functionalization via plasma treatments or silane coupling agents to promote superior adhesion with conductive inks and electrodes. Additionally, copolymerization with conductive additives may elevate the electrical conductivity and charge transport efficiency, enabling reliable operation in sensors, energy storage systems, and stretchable devices under prolonged dynamic stress [14].

As a printing substrate, PLA reinforced with cellulose microfibers or chitin nanofibers can be used, providing increased mechanical strength [15]. At the same time, cellulose acetate exhibits thermal stability and metal adhesion, making it suitable for flexible boards and low-temperature soldering [16]. Moreover, PHB-based composites with wood fibers or carbon nanotubes are used in dielectric or conductive layers due to their improved thermal stability and electrical conductivity [15,17]. In the production of sensors, biobased polymers, such as PLA, chitosan, and nanocellulose, are exploited because of their biocompatibility, contaminant absorption ability, and sensitivity to chemical signals [10,15,17,18,19].

This review aims to offer a holistic perspective on the usage, role, future outlook, and challenges of biobased polymers in PE. Firstly, an analysis of the general properties of biopolymers and the effect of various printing techniques in their processing is presented. Subsequently, various types of biobased polymers and their applications are explored, including polymer blends, copolymers, substrates, conductors, semiconductors, and their integration into organic bioelectronics. The review concludes by highlighting key technical and economic challenges, while also outlining future directions for advancing sustainable electronic technologies.

## 2. Printing Techniques

**Figure 2:**
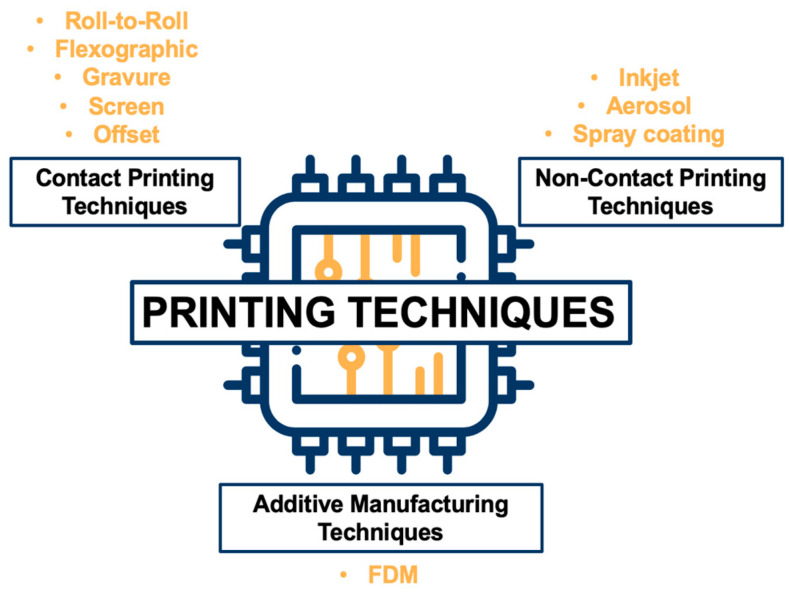
Overview of various printing techniques.

The rapid evolution of PE technology has led to the need to develop and optimize printing techniques to meet the requirements of modern industry. The selection of the appropriate printing technique is a critical factor, as it affects the accuracy, resolution, compatibility with the materials used, as well as the efficiency of the production process. Particular attention should be paid to the relationship between the printing technique and the characteristics of the substrate, especially when biobased polymer substrates are employed. In the context of printable electronics technology, it is worth considering the main categories of printing techniques (contact printing, non-contact printing, and additive manufacturing printing), which enable direct deposition on the substrate. Each of these is characterized by different operating principles, performance, and fields of application, offering alternatives depending on the requirements of each application. Figure 2 summarizes the categorization of these techniques and serves as a conceptual map for the further analysis that follows.

### 2.1. Contact-Based Printing

#### 2.1.1. Roll-to-Roll

Roll-to-roll is a crucial production method in PE technology, as it allows mass and continuous printing on flexible substrates, rather than being a separate printing technique. It acts as a platform on which various printing methods, such as gravure, flexographic, and inkjet, can be applied, enhancing efficiency and industrial-scale production capability [20]. Desired lines or designs are drawn and formed on a printing plate, which is often mounted on a roller and transfers ink to the substrate on contact. The pressure needed is applied either directly or indirectly, through an intermediate roller. Thus, in roll-to-roll printing both additive and detachable processes can be used to fabricate complex structures in a continuous manner. This method differs from traditional manufacturing due to its speed, lower energy consumption, and reduced production costs on a large scale [21].

#### 2.1.2. Screen Printing

**Figure 3:**
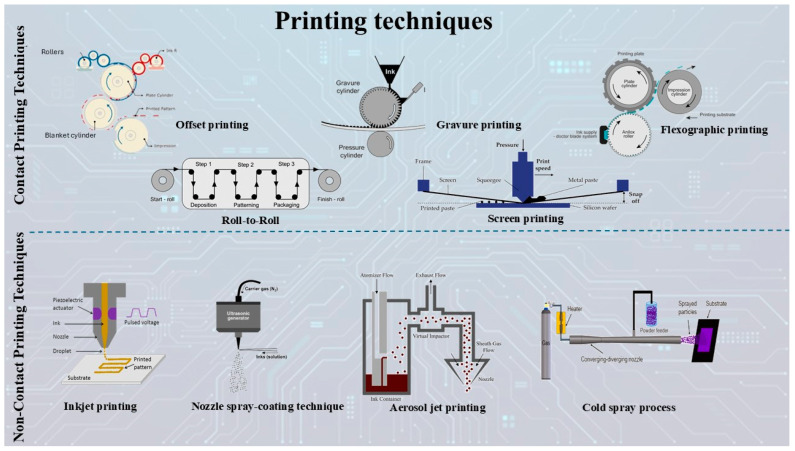
Schematic representation of the main contact and non-contact printing techniques used in PE.

Screen printing is a simple, fast, highly productive printing technique and is the oldest and most widely used method in the industry [2,20,21,22,23]. Screen printing is based on three basic components: the screen, the spatula, and the substrate, which is mounted on a mobile platform. The screen is a reticulated mesh that acts as a mask, with transparent and opaque areas that define the print pattern. A paste (high-viscosity ink) is evenly spread on its surface by means of a flood bar. A spatula is then moved over the screen, applying controlled pressure so that the paste passes through the open areas of the grid and is deposited on the substrate, forming the desired pattern (Figure 3) [1,2,20,21,22,23]. The areas of the grid that are not to be printed are covered with a stencil that defines the image and acts as a printing plate [2,21]. The process is completed with the drying stage, which is usually carried out by evaporation, oxidation, or ultraviolet (UV) curing, and can be accelerated by the use of hot air [2]. It enables rediscovery with consistent results when process constraints are met and solutions are optimized [1].

The quality of screen printing affected and depended on several factors, including the starting material, the density, thickness, and size of the mesh, the printing speed, the withdrawal distance between the screen and the substrate, the viscosity of the solution, and the angular geometry of the spatula [1,2].

Compared to other printing technologies, screen printing presents significant advantages, making it an attractive option for a great variety of applications. In particular, the overall cost of screen printing is low considering both equipment and paste materials. Screen printing offers the possibility of printing on flat or curved substrates, even on the delicate ones, thanks to the flexible nature of the screen, while at the same time it can easily deposit thick films (above 10 μm). The technique provides better control of paste movement due to its high viscosity, and it enables the use of small solid particles—such as metals, carbon, and polymers—which are commercially available and suitable for the manufacture of electrical components [20].

However, like any technology, screen printing has some disadvantages. In particular, high-viscosity inks or pastes require the addition of special additives to enable the functional materials to be formed into a form suitable for printing. The presence of these additives, however, may adversely affect the electrical properties of the materials [20,23,24]. In addition, the final surface of the printed patterns often exhibits increased roughness, due to high viscosity [20,24]. At the same time, the limited chemical resolution of the method acts as a hindrance for applications requiring the printing of detailed and highly accurate structures, as is the case in many modern electronic devices [20].

#### 2.1.3. Gravure Printing

Gravure printing (GP) is a well-known additive manufacturing technique, which can produce high-quality electronic structures with low cost and very high speed (up to 0.1 m/s). This technology is suitable for printing with low-viscosity inks, and the images printed are extremely stable and reliable due to the durability of the image [1,2,24].

GP technology can work in two basic ways, either web-fed printing or sheet-fed printing, depending on the type of substrate and the production scale. In web-fed printing, the rolling mechanism that is used allows printing of a continuous roll of substrate at high speeds. The latter makes it particularly suitable for mass industrial production, offering stability and high quality in the final print. On the other hand, sheet-fed printing relies on sequential sheet feeding, which favors small batch production or use in research and laboratory environments where flexibility and control are more critical than speed [20].

The platen printer consists of the platen roller (or plate), the ink supply unit, an ink removal blade to remove excess ink, and the printing cylinder [1,20]. The scratch roller is the key element of the process, as it bears incised patterns in the form of microcells or ‘teeth’ [1,20]. It is made of a steel base, which is coated with electrolytic copper and polished to protect it from wear and scratches. The geometry of the microcapsules, particularly the width-to-depth ratio, affects the transfer of ink [1,20].

During operation, the scratch plate is immersed in the ink, and the excess material is removed by the doctor’s blade, which is positioned at an angle and exerts controlled pressure for uniform contact. The ink remaining in the slots is transferred to the substrate by means of the contact roller, due to adhesion forces between the ink and substrate [20]. Ink transfer is often improved by using an electrostatic assist system (ESA), which creates an electric field in the contact zone, helping to lift the ink from the cells to the substrate [2]. Precise adjustment of the pressure and angle of the blade is critical to avoid premature wear of the roller by particles or debris that may become trapped under the blade [1]. The ink layer is finally dried by evaporation of the solvent through hot air [2] (Figure 3).

There are many other factors that affect the quality of GP, such as print pressure, blade angle and pressure, speed, proper spacing between cells and dimensions of features in the roller cells, shear force in the printing mechanism, substrate properties (smoothness, ink permeability and porosity, compressibility, water repellency, etc.), and ink properties (ink chemistry, rheological behavior, viscosity, solvent evaporation rate, and curing process) [24].

GP is notable for its ability to use low-viscosity inks, which allows direct printing of high-purity materials without needing extra substances. On top of that, this technology supports high-speed printing, particularly in roll-to-roll layouts, making it ideal for mass production. The microstructure surface of the grass roll allows precise adjustment of the volume of ink transferred, and through the customization of the size and depth of the cells, printing with differentiated material thicknesses can be achieved. Τhe durable metal plate of the gravure cylinder has great durability, maintaining the quality of the printed pattern even after millions of repetitions [20].

Despite the advantages of GP, the method has significant limitations that affect its use in modern applications. First and foremost, the image formation process relies on discrete microcells, which results in a “jagged” appearance when printing straight lines and limits resolution, making it less suitable for high-precision applications. In addition, managing the movement of ink on the substrate surface requires careful control of both ink and substrate properties in order to achieve uniform coating. A major disadvantage is the high cost and prolonged manufacturing time of graphite rolls, which hinder the overall applicability of the method in small batches or prototypes’ production. The overall benefits of the method are also limited by the need for high-contact pressure combined with the rigid nature. This applies especially in uses including flexible substrates, while direct printing on rigid materials becomes problematic. Although alternative approaches have been reported, such as the use of polymeric flexible plates for printing on ceramics with improved resolution (up to 10 μm), these methods remain at an early stage and are not yet widely applied [1,2,24].

#### 2.1.4. Flexographic Printing

Flexographic printing (FP) uses soft and flexible printing plates or a cylinder with design areas where the pixels are raised relative to the rest of the plate surface [2,20]. The main element that controls the transfer of the precise amount of ink to the flexographic plate is the anilox roller, which has small, evenly engraved cells on its surface. The anilox cylinder is coated with ink from the reservoir and the excess ink is removed with the aid of a doctor’s blade. In this way, the ink is retained only within the cells of the anilox cylinder. The ink is then transferred to the raised pixels of the printing plate. The transfer of the ink to the substrate takes place in the pressure zone (nip), where the pressure allows sufficient contact and transfer of the ink. Upon leaving the nip zone, the ink layer separates, with a portion of it being transferred to the substrate. The ink dries by evaporation of the solvent, which is achieved by injecting hot air into the surface (Figure 3) [2,24]. FP plates are mainly made of rubber or photopolymer. The rubber can be etched manually or by laser, while the photopolymer hardens in areas exposed to UV light, with unexposed areas removed by washing [20].

Inks used in graphite printing include solvent-based, UV-sensitive inks that are polymerized by UV radiation, as well as chemically curable inks (thermal or chemical reaction type), which allow the creation of high-resolution patterns on various surfaces [20].

What makes FP advantageous is its compatibility with ink of relatively low viscosity (50–200 cP), which facilitates ink formation. The flexographic plate is flexible, low cost, and easy to manufacture, while its elasticity makes it capable of printing ink on a wider range of substrates, including rigid or rough surfaces. The low pressure used during printing allows the application of thinner layers of ink. The ability to print thinner layers more accurately is directly related to the ease of achieving clean and precise lines, which makes flexography ideal for applications requiring high detail and print quality on a variety of substrates [20].

Although FP is widely used because of its simplicity and suitability for flexible substrates, it nevertheless presents technical limitations that restrict its application in demanding fields, such as printing functional electronics. The resolution achieved usually ranges between 50 and 100 μm, with the potential for improvement down to 20 μm only under strict control of the process parameters. However, physical wear of the cells on the printing plate and pressure from the contact roller can cause alterations in the geometry of the patterns, leading to non-uniform or discontinuous patterns. In addition, the need to deposit thicker layers requires repeated printing cycles with high-precision alignment between passes, increasing procedural complexity. The FP method also exhibits a weakness in achieving high electronic performance, as it does not allow for the creation of detailed patterns with fast response and low power losses. This often leads to degradation of critical operational characteristics, such as load carrier mobility and line capacity. In addition, surface and edges’ irregularities, pores, and limited availability of suitable functional materials exacerbate the accuracy and stability problems. Overall, despite its convenience and widespread use, FP presents several limitations that necessitate further optimization of the technology for high-precision applications [1,20].

#### 2.1.5. Offset Printing

Offset printing is an indirect printing method in which the ink is first transferred from the flat printing plate to an intermediate blanket and then to the substrate, usually paper, through the nip. The plate is chemically modified to carry virtual (hydrophobic and oily) and non-virtual (hydrophilic) areas, which manage ink and water adhesion, respectively. The wetting rollers apply a thin layer of moisture to the non-virtual areas to prevent unwanted ink deposition, while the ink rollers cover the virtual areas with an ink film. The process is wet-on-wet, i.e., the color inks are applied sequentially without intermediate drying, which leads to a partial transfer of the ink and to the blanket of the next color stage. After final printing, the ink dries through absorption by the substrate or through physical and chemical mechanisms, such as evaporation, oxidation, or polymerization, ensuring the final stabilization of the image. The accuracy of the method is based on the control of the surface energy and the fine balance between ink and moisture [2,20]. Unlike conventional offset printing, which relies on the use of a moisture solution, waterless offset printing technology is a more recent development. In it, the entire printing plate is covered with a thin, waterproof layer of silicone in the non-virtual areas, while the layer is selectively removed from the virtual areas, making them receptive to ink. This eliminates the need for a wetting unit, which simplifies the equipment and enhances the accuracy of ink deposition. Ink remains only in the patterned areas, moving away from the rest, thanks to the hydrophobic protection of silicone [20] (Figure 3).

As an evolution of traditional methods, reverse offset printing (ROP) is emerging as a promising process for achieving high-resolution printing, especially in the field of PE [25], leading to a remarkable reduction in material usage. ROP methods allow miniaturization of printed features down to micrometer sizes, producing conductive structures [26]. The forming principle is converted from wetting to adhesion and fracture, allowing avoidance of resolution problems encountered when forming liquid inks (e.g., spreading, splitting, coalescence, bumps, and brown ring shapes) [25]. The ability to form thin layers with near-rectangular side profiles that closely match the desired layout dimensions of the design is a unique advantage of ROP for manufacturing multilayer layouts, while offering good uniformity over a large area [26]. Finally, it enables high resolution, dimensional accuracy, and is a scalable, low-cost, low-temperature forming method [27]. There are several types of ROP systems, such as roll-to-sheet (R2S), sheet-to-sheet (S2S), and roll-to-roll (R2R). A variety of inks have been used for ROP, including gold, silver, and nanowire nanoparticles, copper nanoparticles, palladium nanoparticles, indium tin oxide nanoparticles, poly(3,4-ethylenedioxythiophene):poly(styrene sulfonate), dielectric materials, organic semiconductors, etching resistors, quantum dots, black/color dyes for color filters, solder/flow, and metal oxide precursors. ROP has been applied in various fields, such as color filters, thin-film transistors, sensors, printed memories, touch screens, etching resistors, organic light-emitting diodes, quantum dots, metamaterial absorbers, antennas, electrodes for photovoltaics, protein modulation, and seed coatings for chemical and electroplating [27].

### 2.2. Non-Contact-Based Printing

#### 2.2.1. Inkjet Printing

Inkjet printing is a relatively new technology that has attracted considerable scientific interest and is still in an early stage of development [24]. It is a digital printing method that does not require the use of a matrix or mold to transfer the image [20,22], but uses precise control of ink droplets, which are ejected from computer-guided nozzles, creating digital designs using a droplet matrix [1,20]. The image is stored digitally on a computer and, using the special software that accompanies the printing equipment, it is possible to create, edit, and print the files. The print heads, on which the nozzles are mounted, can be either fixed, wide format, offering high speeds, or mobile, scanning the surface of the material [2,20]. Inkjet technologies are divided into two main categories based on the principle of operation: continuous inkjet printing (CIJ) and drop-on-demand (DOD; Figure 3).

In CIJ printing, ink droplets are continuously produced and pass through charging plates, acquiring an electrical charge and being guided toward the substrate by an electric field. The unwanted droplets are diverted and recycled back into the ink reservoir. In contrast, in DOD technology, droplets are ejected only when required, by applying a voltage pulse. This method allows for more precise control of the amount of ink but carries the risk of nozzle blockage due to solvent evaporation when the print head is idle [28]. There are two main mechanisms for ejecting droplets from the head: thermal propulsion (bubble jet) and piezoelectric propulsion. In the first case, a heating element creates a bubble that expels the droplet from the nozzle. In the second, a piezoelectric ceramic element deforms when a voltage is applied, pushing the droplet toward the substrate [21]. The drying process in inkjet technology is a critical stage for the stabilization and functionality of printed materials. Drying can take place through simple evaporation of the solvent, through chemical reactions—such as polymer cross-linking—or by crystallization of the active ingredients. Depending on the material and the application, post-processing, such as thermal annealing or fusion, may also be required to achieve the desired mechanical, electrical, or optical properties [23,24]. The most common image file format for inkjet printers is the monochrome bitmap, where black pixels correspond to print areas and white pixels to non-print areas. The printer software converts the bitmap into electrical pulses that trigger the printer head to fire the ink. Alternatively, the print pattern can be set manually through parameters such as droplet spacing and lines, especially for simple designs [20].

Inkjet technology offers a flexible and cost-effective method for creating and printing a wide range of patterns, making it particularly suitable also for high-precision applications. Thanks to its ability to print directly on a wide variety of substrates—such as glass, plastic, fabric, and paper—and to use low-viscosity inks without binders, it achieves nanometer-level thicknesses with minimal material waste. The process is simple, requires few steps, and is environmentally friendly, while supporting personalized and on-demand printing without the use of molds. While the speed is limited, the print quality is extremely high, which makes it suitable for hybrid applications in combination with conventional techniques [23,24].

Despite being flexible and accurate, inkjet technology is often limited by some drawbacks. For instance, the process is relatively slow due to raster movement and drop-by-drop printing. Nozzles are prone to clogging and careful ink adjustment is required (viscosity, surface tension, and humectants). In addition, pickling phenomena and deviations in the launch angle are observed, which affect accuracy. Scaling at the industrial level requires multiple heads, which increases complexity and the risk of errors [22,24].

#### 2.2.2. Aerosol Jet Printing

Aerosol jet printing (AJP) is a direct contactless printing technology developed by Optomec [1,20], which is based on a different operating principle to inkjet printing. The ink, which may include solutions or suspensions of nanoparticles (such as metals, alloys, ceramics, polymers, adhesives, or biomaterials), is converted into an aerosol with droplets of 1–5 microns in size by a pneumatic or ultrasonic process using a nebulizer (Figure 3).

The aerosol is directed toward the nozzle and concentrated by means of a ring layer of compressed air, forming a high-velocity flow that is sprayed onto the substrate. At the same time, the nitrogen flow and the envelope gas stream concentrate and direct the aerosol flow into the substrate [24]. The pattern is formed by continuous lines, with the nozzle remaining stationary while the sample surface is moving [20]. AJP technology offers significant advantages, such as high resolution (up to 10 μm), compatibility with a wide range of inks and substrates, even on non-flat or curved surfaces, and the ability to print complex, 3D patterns through precise flow control. Thanks to its low process temperature and absence of blocking problems, it is a reliable choice for functional materials [20,24]. However, the technology has a limited speed (up to 12 m/min), it is not suitable for low-boiling-point solvents, and it requires expensive equipment. Also, phenomena such as droplet cloud formation or local crystallization may reduce print quality. The need for constant air flow makes dynamic parameter adjustment difficult and the complexity of the system limits the possibility of mass production [21].

#### 2.2.3. Spray Coating

Spray coating (SC) is a highly efficient technique used to apply solutions or materials to surfaces by spraying, creating a uniform layer on the substrate. This method is based on the dispersion of the liquid solution on the surface of the substrate by aerosol or spray application [22,29]. The SC technique is distinguished by its low cost, ease of application, and ability to control the thickness of the coating. It is a preparatory method that acts as an intermediary between laboratory development and large-scale industrial production. In addition, it is suitable for the deposition of low-viscosity materials, allowing the creation of thin and uniform coatings, which are crucial for the reliability of electronic systems. Mechanical simplicity, design flexibility, high production speed, and the ability to apply materials to non-traditional substrates make the SC method particularly attractive for applications in flexible hybrid electronics, combining functionality, adaptability, and industrial feasibility [22].

While the SC technique is distinguished by its general advantages, more specific applications, such as cold spray (CS), offer additional possibilities, such as solid-state surface metallization. It is based on the impact of high-velocity particles (typically micron-scale metallic particles) on a target surface at low temperatures. In this technique, as shown in Figure 3, particles are accelerated to high velocities through a converging–diverging nozzle and then collide with a target surface. When the impact velocity of the particles on the target exceeds a critical value, they undergo plastic deformation (bonding), resulting in a functional coating/print of high strength and density. The high-impact velocity facilitates self-adhesion of the particles to the target surface, leading to a coating with strong adhesion due to the dissipation of the particles’ kinetic energy on the substrate surface [30,31].

This technique is a promising approach for rapid, scalable, and cost-effective mineralization. One of its key advantages is its low process temperature, which allows the use of heat-sensitive substrates. It offers strong adhesion strength, creating coatings with excellent adhesion to the substrate, while also featuring a high deposition rate, enabling rapid and high-performance deposition of materials. Unlike many traditional methods, the process can be performed without the need for a mask and vacuum, in ambient conditions. In addition, it demonstrates compatibility with various polymer substrates, such as polyethylene terephthalate (PET), polyethylene naphthalate (PEN), kapton (polyimide), acrylonitrile butadiene styrene (ABS), polyether ether ketone (PEEK), carbon fiber reinforced polymer (CFRP), and polytetrafluoroethylene (PTFE), and offers direct deposition capability, allowing for the immediate creation of conductive traces. A major advantage is that it does not require post-sintering, which is often needed in other methods to improve conductivity and adhesion, but it does increase cost and can cause oxidation, making the technique suitable for low-thermal-budget substrates [30,31]. Of course, its low spatial resolution compared to other printing techniques is a disadvantage that does not, however, significantly limit its use in low-volume applications [30]. It is used extensively in the production of flexible electronics, surface metallization, millimeter-scale electrode fabrication, flexible microelectronics, and flexible microheaters [30,31]. In addition, it can be applied in the manufacture of light-emitting diode (LED) circuits, repair of damaged field-effect transistors (FEs), and multi-material electronics [30].

In addition to the CS technique, another specialized variant of the spray method that is of particular interest for high-precision applications is the ultrasonic spray coating technique. The ultrasonic nozzle spraying (US) method is widely used for the fabrication of thin nanocoatings, and its usefulness is also being extensively investigated in the fabrication of organic electrodes. It is based on the dispersion of the spray ink through high-frequency vibrations induced in the spray nozzle and causing atomization [32,33]. High-frequency mechanical vibrations, generated by piezoelectric transducers, are applied to the tip of the nozzle to create capillary waves within the liquid film, and once the amplitude of the capillary waves reaches a critical value, the waves become too high to support themselves and tiny droplets fall from the tip of each wave, thus atomizing the feed solution. The oscillating US breaks down the solution (ink) to form micron-sized droplets, which are delivered by spraying them onto the surface of the substrate (Figure 3) [33].

Spraying by the US method consists of extremely small droplets of solution, a few micrometers in diameter, which allows the production of uniform organic films. This uniformity is maintained over time, as the ultrasonic mechanical vibrations of the nozzle contribute to the stability of the solution composition and spraying conditions. At the same time, the operating principles of the US technique offer significant advantages, such as minimizing nozzle clogging and solution waste, precise control of spraying at low speed or flow, and overall process stability. For all these reasons, the US spray technique is proving to be an effective, reliable, reproducible, and promising printing technique [32,33].

### 2.3. Additive/3D Printing

#### Fused Deposition Modeling (FDM) 

**Figure 4:**
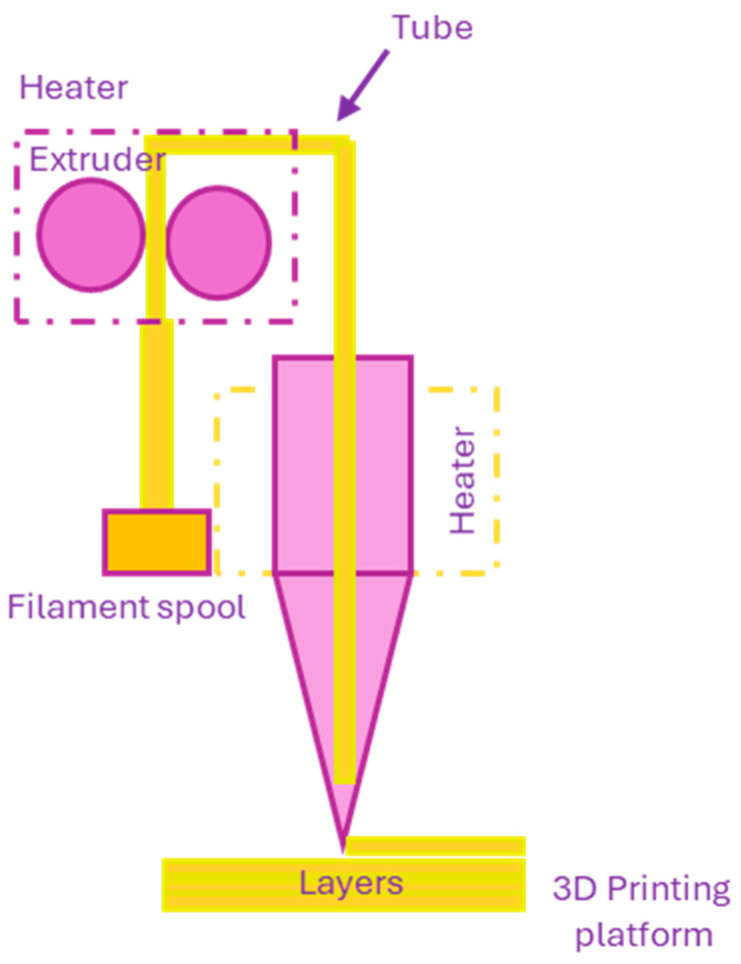
Schematic diagram of FDM technology [37].

Fused deposition modeling (FDM) technology is one of the most popular 3D printing methods used in the field of additive manufacturing [34,35,36]. Developed in 1988 by Steven Scott Crump and commercialized by Stratasys in 1992 [35], the basic mechanism involves taking a thermoplastic material, melting it, and extruding it through a heated print head. The material is ejected onto a printing surface, which is usually a mesh (e.g., glass or aluminum), in thin layers that are stacked on top of each other to form the final structure [34,35,36,37]. The thermoplasticity of the polymer allows the layers to be bonded together during printing and fixed at room temperature after printing [36]. This method allows the creation of complex geometries without the need for molds or additional tools (Figure 4) [34,36].

The most common materials used in FDM techniques include PLA, ABS, polypropylene (PP) [34,35,36], poly(ethylene terephthalate) (PET), Nylon 6, and thermoplastic polyurethanes. The FDM method is highly cost-effective, both due to low installation costs and low material consumption, making it ideal for small businesses and personal use. The process is easy to install and maintain, allowing its use in a variety of sectors without complex procedures. In addition, FDM enhances the production of complex geometric shapes without requiring increased costs, unlike traditional production methods [35]. The quality of the printed part in the FDM technique depends on the fillet used. The fillet, made of thermoplastic materials, should maintain a constant diameter, as variations can cause jamming or uneven firing. In composite fillets, reinforcements are added for better strength and thermal resistance. The key parameters of the FDM process include build orientation, layer thickness, air gap, raster angle, raster width, contours, and contour air gap. Correct setting of these parameters is critical to the quality of the print [34]. The FDM printer uses various printing factors, such as the printer temperature, the diameter of the print head, the angle of the print pattern, the thickness of the layers, and the filling strategy of the inside of the printed object. The correct setting of these parameters is critical to the quality and mechanical properties of the final product. In terms of print quality, layer resolution, raster ratio, and nozzle and platform temperature are also critical for layer bonding and accurate material flow [37]. During FDM printing, problems such as platform misalignment, which causes distortion or failure to adhere layers, nozzle blockage, which interrupts material flow, inability to adhere to the frame, and material flow disruption, which leads to incomplete printing, can occur.

To avoid these problems, proper alignment and cleaning of the platform, use of quality material, and adjustment of printing parameters (temperature, speed, and nozzle distance) should be ensured. This helps to avoid errors, such as stringing, and ensures uniform printing [35,37].

## 3. Biopolymer Composites in Electronics

The ever-increasing technological development has significantly affected every aspect of society, making people increasingly dependent on the electronics sector to meet their daily needs, as well as to achieve a more comfortable and smarter lifestyle. The latter raises strong environmental concerns about the management of e-waste. The constant rise in the production and consumption of electronic devices combined with the amount of discarded electronics, often containing non-degradable polymers and toxic chemicals, is increasing significantly [38]. Traditional petroleum-derived polymers, unlike many natural materials, present challenges in terms of biodegradability and contribute to the growing e-waste problem. ‘Green’ electronics research, inspired by the design of natural bio-composites, therefore, emphasizes shifting from petroleum-derived polymers to bio-derived polymers to achieve enhanced biodegradability and sustainability and reduce e-waste [39,40].

The search for alternative materials with environmentally friendly characteristics is, therefore, becoming imperative. Biopolymers, which are essentially polymeric biomolecules derived from living organisms [41], are the focus of research interest, in particular chitosan, cellulose, alginic acid, and PLA and their biopolymer composites, due to their special physicochemical properties and their potential to be exploited in 3D printing technologies and to be integrated in a variety of electronic applications [38,42,43].

### 3.1. Biopolymers

#### 3.1.1. Chitosan

**Figure 5:**
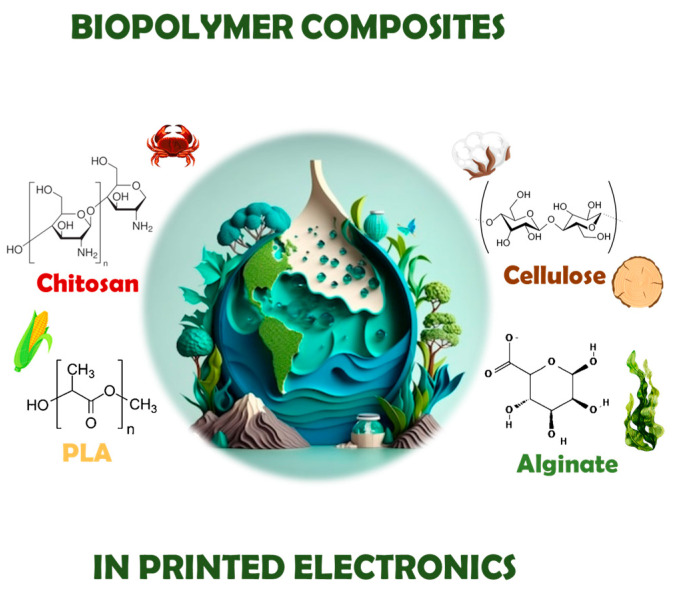
Schematic representation of biopolymer composites in electronics.

Chitosan is ranked as the second most abundant natural biopolymer after cellulose. It is a derivative of chitin and is obtained by deacetylation of chitin, which is found in the exoskeleton of crustaceans and insects, as well as in the cell walls of fungi and yeasts [20,36,41,42,43,44,45,46,47,48,49]. Its molecular structure consists of D-glucosamine and N-acetyl-D-glucosamine units with β(1–4) bonds, as presented in Figure 5 [44].

Generally, polysaccharides (chitosan, cellulose, alginate, etc.) often impose several limitations regarding demanding applications, such as printed electronics. They are characterized by low intrinsic electrical conductivity, therefore requiring the incorporation of conductive fillers or ionic salts to achieve electrical functionalization. The additives utilized, however, may result in deterioration of the material’s mechanical properties, such as its integrity and stability. Moreover, many native polysaccharides inherently exhibit low tensile strength, decreased elasticity, and limited durability under repeated deformation. Additional practical challenges of biobased materials include the reproducibility and scalability toward their broader implementation. The variability in biological resources can impact batch-to-batch consistency, increase purification-related costs, and necessitate specialized fabrication techniques for uniform thin-film or intricate patterns’ formation [50].

Specifically, chitosan has been described as a material with insufficient mechanical properties, poor water resistance, and inferior thermal stability compared with the requirements for printed electronics applications [51]. It also becomes highly brittle due to its elevated glass transition temperature, while its increased hydrophilicity promotes pronounced swelling and, consequently, a reduction in structural integrity. Moreover, according to the literature, the ion exchange capacity of pure chitosan could be further enhanced [52]. To address the aforementioned drawbacks, for applications including printed electronics, chitosan often serves as a composite constituent along with co-components. For example, inorganic materials are frequently incorporated as additives [43].

Among its advantageous properties, chitosan is a biodegradable, non-toxic, and non-allergenic material [43,45,47,53] and is characterized by excellent biocompatibility, chemical stability, and the ability to form a transparent film, which makes it suitable for use as a dielectric substrate [38,43,45,46]. The highly reactive hydroxyl groups and amino groups (-OH and -NH_2_) contained in its structure allow it to form strong interfaces with the fillers (either at the microscale or nanoscale) [43,46,47,49] and, especially when combined with high-conductivity materials, such as poly(vinyl alcohol) (PVA) or carbon nanotubes, make it suitable for use in bioelectronic applications, such as conducting polymers for sensors and transient electronic devices [38], enhancing electromagnetic shielding capability with an efficiency of 25–60 dB. Its low permeability to methanol [43,44,46], its amphiphilic character [43,46], high thermal stability, and its ability to act as a moisture barrier further support its use as a solid polymer electrolyte [43] (mainly chitosan-poly(vinylphosphonic acid) (PVPA) composite membranes [44]) in electrolyte-based fuel cells [43]. Its low crystallinity helps to enhance its mechanical strength and elastic modulus, enabling its use in transparent, flexible electronics, logic circuits, distortion sensors, and diodes [43,46]. Owing to its insulating properties and its low molecular weight, chitosan significantly expands its applications in the manufacture of supercapacitors, disposable transistors, and biosensors [43,46,47], while it is used to create solid or semi-solid electrolytes with the addition of ionic liquids, such as 1-ethyl-3-methylimidazolium thiocyanate, significantly increasing the ionic conductivity of the mixture [53]. It exhibits excellent swelling capacity and adjustable drug release, which are exploited in biomedical electronic devices due to the ability of chitosan to form hydrogels by cross-linking with glutaraldehyde [54]. Chitosan/graphene/gold (AuNP) composites have yielded excellent electrochemical responses as glucose biosensors. In addition, modified derivatives, such as carboxymethylated chitosan incorporating palladium nanoparticles, have demonstrated the potential to enhance functionality and protection in advanced electronic applications [47,49].

#### 3.1.2. Cellulose

Cellulose is a homopolysaccharide and is the most abundant organic polymer in nature, accounting for about 90% of the composition of cotton and 50% of wood [22,38,43,45,46,49,53,55]. Its wide availability, combined along with its low cost and low molecular weight, make it a highly attractive material and key element in the development of bio-composites in electronics [38,43,46,47]. Its structure consists of linear chains of glucose molecules joined repeatedly by β-1,4 glycosidic bonds [43,46,47,49].

Cellulose is used extensively as a biodegradable substrate in many electronic applications, such as printed circuits and nanogenerators [43,45,49]. In addition, it is used in biosensors, barrier films for electronic devices, and as a reinforcement in composites for greater thermal stability and durability. It is also used as a carrier in drug delivery systems for intelligent electronics, exploiting its unique properties to build advanced, sustainable technologies [43].

Nevertheless, unmodified cellulose’s properties are not considered optimal. For example, its decreased solubility in conventional solvents limits its processing and formulation options, necessitating specialized equipment and chemical treatments. Furthermore, cellulose is generally regarded as a hard substance with good mechanical strength, however, its mechanical performance in certain applications is still notably inferior compared to synthetic materials. In addition to the above, its hydroscopic behavior can result in reduced dimensional stability and susceptibility to expansion or contraction in response to humidity fluctuations. According to the literature, cellulose also displays electrical limitations, mainly due to its inherently decreased ionic conductivity, which constrains cellulose’s employment in electrically functional applications. On the same note, the necessity to gain deeper insight into the gap area of cellulose for electronic applications has been underlined. The gap area constitutes the void spaces between cellulose fibers, and it markedly influences both mechanical and electrical performance. Despite the above drawbacks and although cellulose extraction involves operational complexity and substantial energy demands, its advantages remain predominant [56]. Additionally, since it has been described as a material difficult to monitor, modifications or blends are often used [45].

Forms and derivatives of cellulose are cellulose esters (cellulose acetate), cellulose ethers (carboxymethyl cellulose and hydroxyethyl cellulose), and cellulose nanofibers (nanocellulose) [45]. The use of cellulose-based blends, their composition, and their role in biobased electronics applications are further discussed in detail in Section 3.3. It includes CNCs (cellulose nanocrystals), CNFs (cellulose nanofibrils), and MCC (microcrystalline cellulose) [55]. These materials provide a fully biobased platform for developing advanced electronics. In addition, the use of cellulose nanopaper in organic field-effect transistors (OFETs) offers high dielectric stability and transparency, making the material suitable for applications in low-voltage systems [47,49]. Operating electronic devices at low voltage (<2 V) is a strategy to save energy and reduce costs. For this reason, organic field-effect transistors with ionically conductive nanocellulose “paper” have been developed and exhibit high optical transparency (>80%), superfine surface area, high ionic conductivity, dielectric stability, and thermal stability, making them highly suitable for low-voltage applications in electronic systems [43,49].

Regenerated cellulose, such as the NatureFlex™ film form, is an alternative with excellent biodegradability and satisfactory mechanical strength. Although its surface shows relatively high LED roughness, it has high transparency and satisfactory thermal resistance. The addition of a heat-seal coating improves the adhesion of the printed conductive paste, offering excellent performance as a substrate for various applications. Bacterial cellulose (BC), which is produced by bacteria and has a similar chemical structure to plant cellulose, is considered to be the purest form of cellulose. Thanks to its high crystallinity, tensile strength, and unique fibrous structure, it has been used neat and in composite form for advanced electronic applications [43,46,47,49]. In addition, BC can withstand high ultrasonic wave speeds with minimal energy loss, making it ideal for the fabrication of Hi-Fi devices, such as headphones, speakers, and microphones. BC’s three-dimensional fibrous structure also makes it suitable for flexible, high-performance nanoelectronics, such as molybdenum disulfide (MoS_2_)-based phototransistors, which are grown on transparent, eco-friendly nanofiber cellulose (NFC) substrates [43].

In parallel, printed dipole antennas have been implemented on flexible cellulose substrates, which operate efficiently even at high frequencies (8–12 GHz). This highlights the potential of cellulose to support demanding electronic systems while maintaining their flexibility and biocompatibility [22]. Various forms of cellulose and its composite materials are summarized in Figure 5.

#### 3.1.3. Alginate

Alginate is a natural anionic polysaccharide that is extracted from brown algae [43,45,46,47,49,54] and contains carboxyl and hydroxyl functional groups, allowing easy mixing with other materials and the formation of composites with other polymers or fillers. Its molecular structure includes β-1,4 bonds between D-mannuronic and L-glucuronic acid, which endows it with remarkable gel-forming properties [43,46,47,49]. While alginate is known for its biocompatibility, non-toxicity, and high water absorption (up to 300 times its weight) [44], this latter feature is not considered favorable for printed electronics, since it can promote swelling or deformation, resulting in temporal variations in dimensions, dielectric behavior, and mechanical integrity [50]. Nevertheless, the high gel-forming ability of alginate supports its application in biosensors in the health sector [43,46]. It should be noted, however, that ionically cross-linked alginate gels exhibit limited long-term stability, particularly in physiological conditions, due to their tendency to exchange their divalent ions with monovalent cations from the surrounding medium [51]. Moreover, it has been stated that modifications of alginate intended to adjust its conductivity often influence the material’s surface chemistry and charge, thereby requiring careful evaluation for applications related to biological systems [50] (e.g., biosensors).

Additional limitations of alginate that should be considered for printed electronics applications are its insufficient mechanical strength, inferior barrier properties, limited compatibility with heavy metals, and instability under thermal processing. The most prominent solution in order to overcome these problems constitutes the combination of alginate with different biopolymers or synthetic polymeric materials [57].

Nevertheless, alginate still offers notable benefits for applications in printed electronics, since it exhibits excellent proton conductivity (~5.5 × 10^−3^ S/cm) and the presence of active sites in its dense functional molecules makes alginate an ideal candidate for the development of materials with excellent bilayer capacitance (~2.0 μF/cm^2^). These critical properties enhance its potential for advanced electronic applications [43,46,47,49].

#### 3.1.4. Poly(Lactic Acid)—PLA

PLA is a recyclable thermoplastic [42,43,44,45,46,47,55,58] polyester belonging to the synthetic biopolymers [38] and is produced by polymerization of the condensation of lactic acid monomer [43,49]. Lactic acid is obtained mainly by fermentation of renewable plant raw materials, such as sugar cane, cassava, corn, and other starchy plants [38,42,43,45,46,47,49,54,55,59,60,61].

It is one of the most important bioplastics on a global scale [53,60] and thanks to its biodegradable nature, under industrial composting conditions, it has the potential to be processed at low energy cost [38,43,49,59,61].

Its thermal resistance (T_m_ ≈ 150–176 °C) and its high transparency (optical transmission up to 94%) make it particularly suitable for extensive use as a surface substrate in the manufacture of various electronic devices, supercapacitors, 3D printing applications, EMI shielding, and actuators, and due to its good biocompatibility and low toxicity, it can be used in bioelectronic applications, such as pacemakers, brain and heart wave recorders, as well as in nanodiagnostic devices [43,46,47,49,55]. Its special surface allows good adhesion of conductive silver inks, providing good electrical conductivity when used as a substrate in conductive patterns and electronic circuits.

PLA, despite its numerous advantages, presents certain limitations. In more detail, it exhibits inferior mechanical properties, such as elevated brittleness, weak impact resistance, and decreased toughness, and its elongation at break values remain below 10% [62,63,64,65]. Furthermore, PLA is not thermally stable since it presents the ability to deform under relatively high temperatures (heat deflection temperature at 53–56 °C [63], Tg ~ 60 °C). Consequently, in electrical applications involving exposure to elevated temperatures (e.g., motors, lighting systems, and transformers), this limitation may trigger material degradation and failure, adversely affecting the structural integrity and service lifetime of the electrical component. Regarding the electronics field, another major challenge of PLA is its electrical behavior. As an insulator, it exhibits adequate dielectric properties for certain low-voltage applications, but not for high-voltage components, such as cables and connectors, where efficient electrical insulation is critical [64]. At the same time, PLA’s inherent insulating properties, despite their limitations, restrict its suitability for applications requiring electrical conductivity [65]. In addition to the above, PLA is also considered a costly material compared to conventional polymers. Nevertheless, the aforementioned limitations can be addressed by reinforcing PLA through the incorporation of various fillers and additives. For its brittle nature, low resistance to thermal deformation, and relatively high cost [38,42,60], materials such as CNCs, CNFs, and MCC can be employed (Figure 5).

In addition, it can be reinforced with natural fibers, such as wood, straw, or hemp, improving its mechanical properties and thus further extending its range of applications in structural and electronic materials [38,42].

The use of PLA in electronic subsystems is in line with the principles of green chemistry and the circular economy. As a recyclable and biodegradable material, it reduces environmental pollution and dependence on toxic raw materials, while allowing for simplified recycling and reuse of key components [61].

### 3.2. Protein-Based Nanocomposites

#### 3.2.1. Protein-Based Nanocomposites

Proteins are macromolecules whose structure is determined by the sequence of amino acids. These structures define the physical, chemical, and mechanical characteristics of materials, such as their biocompatibility, stiffness, elasticity, and stability. The various functional groups, amphiphilic nature, and flexibility of long chains result in versatile alterations and promote their customized interactions with synthetic materials.

The interest in protein-based bio-nanocomposites derives from the customized nanoscale structure, enhanced mechanical properties, and inherent biocompatibility. The diverse chemical properties of protein materials allow for their easy incorporation with abiotic materials via covalent and non-covalent interactions, including hydrogen bonding, hydrophobic forces, and electrostatic forces [66].

Integrating proteins with various materials can result in enhancement of the device’s sensitivity and conductivity, fostering signal transmission, and improved stability, mechanical strength, and energy density. In recent times, the development of materials science, biochemistry, and nanotechnology has allowed for the modification of protein internal structures through molecular design, heterostructure preparation, and dopant introduction, greatly improving the physical and chemical characteristics of flexible devices [67].

Muñoz et al. [68] developed 3D-printed nanocomposite graphene electrodes (3D-nGEs), made of a commercially accessible graphene/polylactic acid filament, that were covalently biofunctionalized using an extracellular matrix protein (namely, fibronectin). The developed bioelectronic system can potentially function as a useful tool to in situ assess and monitor both adhesion of breast cancer cells and the response of these cells to anti-cancer drug treatment.

#### 3.2.2. Silk-Based Nanocomposites

Natural silk is a continuous long fibroin fiber synthesized by the solidification of the liquid silk secreted by mature silkworms. Silk is renowned for its great mechanical flexibility, biocompatibility, non-toxicity, etc. [69].

Silk fibroin (SF) consists of repetitive amino acid segments with unique functional characteristics, resulting in hierarchical nanostructures with remarkable mechanical properties. Due to these multiscale hierarchical structures, SF presents high strength, stiffness, excellent fracture resistance, and elasticity, when compared to other biopolymers. Given these appealing mechanical characteristics, SF has been combined with diverse inorganic materials to acquire improved properties and broaden its applications [66].

SF presents enhanced biocompatibility, water solubility, and adjustable biodegradability that can range from hours to years depending on the processing methods or the post-processing procedures. Generally, taking into consideration the advantages mentioned above along with the fact that it is a lightweight and transparent material and undergoes simple processing, silk has expanded its application in flexible electronics [70].

Despite the advantages mentioned, silk’s limitations should not be overlooked. Despite its superior flexibility, SF performance is still inferior compared to conventional materials for flexible electronics applications [70]. Additionally, since it has inherently insulating properties, modifications are needed in order to achieve conductivity. For example, conductive layer deposition can be performed on SF surfaces, however, certain problems persist. In more detail, the mechanical durability of the conductive layer is often inadequate, and it consequently exhibits susceptibility to peeling or cracking under constant friction, bending, or tensile stress, ultimately resulting in conductivity decline. Therefore, as stated in the literature, achieving smooth and reliable integration of electronic components with silk-based materials has proven to be challenging in some instances [70,71,72]. Furthermore, SF exhibits brittleness at low water content and limited mechanical strength at high water content [70]. Due to the aforementioned behavior, if a silk-based electronic device is exposed to an aqueous environment, water molecules disrupt the intermolecular hydrogen bonding within the silk structure, resulting in the expansion of the material and the formation of a randomly curled morphology, which causes immediate disconnection of the metallic conductive networks and consequent loss of the device’s functionality [56,71].

Since the inherent or modified properties of SF achieved through physical or chemical methods often fail to meet certain functional requirements, it is commonly combined with secondary components to impart enhanced characteristics [72]. These SF-based nanocomposite structures are made using various kinds of synthetic functional nanomaterials. Because of its numerous chemical functional groups, SF shows robust interfacial bonding with synthetic nanomaterials, preventing undesirable aggregation and providing outstanding mechanical properties. The usual methods for producing SF-based nanocomposites include (1) directly integrating presynthesized nanoparticles into silk solutions for self-assembly into bulk materials, (2) in situ creation of nanomaterials within the SF matrix, and (3) methods involving silkworm feeding [66].

When creating conductive silk materials, the presence of active functional sites within their structures facilitates their integration with different conducting elements, such as conductive polymers, carbon-based fillers, and metallic interfaces, leading to highly conductive silk fibroin complexes. Because of their excellent electronic conductivity, combined with adjustable forms, high mechanical robustness, programmable biodegradation, and non-inflammatory degradation byproducts, the conductive silk advanced materials show promising potential in biomedical applications, such as wearable flexible sensors, electrochemical biosensors, implantable electronics, tissue/bone regeneration, cell scaffolds, and wound dressing.

A typical application of conductive SF materials is the development of a standard pressure and strain sensor. This sensor can monitor a variety of the human body’s movements, such as body motions, facial expressions, and pulse variations. Its function is based on the conversion of mechanical motion into an electrical signal based on a change in electrical resistance or capacitance [71]. Silk-fibroin-based materials can be produced in several forms, such as fibers, films, gels, or 3D porous structures, allowing their use in various applications, including electrodes, power generators, or sensors with high stretchability [73]. Kadumudi et al. synthesized a multifunctional nanocomposite by mixing silk fibroin and reduced graphene oxide. Specifically, this conductive silk–graphene hydrogel has strong adhesive, self-healing, and adjustable properties for sensing physiological data from the body. The addition of the graphene element led to thermal and electrical conductivity of the hydrogel. In addition, the hydrogen and electrostatic bonds among silk fibrin, calcium ions, and the graphene component enhanced its mechanical properties. Taking advantage of these distinctive properties, the authors created a long-lasting and self-healing bionic glove, aiming for the detection of hand gestures and translation of sign language [74].

#### 3.2.3. Collagen- and Gelatin-Based Nanocomposites

Collagens can be classified into the fibrous proteins, and they represent the primary component of skin and bones and have the characteristic triple-helix structures. Given collagen’s biocompatibility and biodegradability, its nanocomposites find primarily application in tissue engineering. In comparison with silk, the use of collagen in electronic-based applications is relatively recent and the research related to it is limited [75]. Collagen-based nanocomposites, due to their biocompatibility, are used in the manufacture of biosensors, EMI shielding, capacitors, biological memory devices, wearable sensors, artificial electronic skin, and other electronic devices [43,76].

Andonegi et al. created collagen films with graphene oxide nanoparticles (GO NPs). The results showed increased thermal stability and enhancement of the mechanical properties due to the addition of GO NPs. Furthermore, an increment in electrical conductivity was observed and, consequently, the development of a natural-based bending sensor for soft robotic applications was achieved [77].

A significant challenge faced with collagen’s utilization is related to its processability and pure water solubility [75]. Due to the variable sources of collagen, differences in the molecular structure, purity, or printability can be detected among the same material category, thereby necessitating tailored processing techniques [78]. Furthermore, despite its typical insolubility in water, collagen’s mechanical properties have been found to alter depending on the humidity conditions [38,79]. Its behavior in wet and dry environments presents substantial changes (e.g., under wet conditions, stress levels at collagen–metal interfaces increased, resulting in enhanced delamination), a characteristic notably challenging since printed electronics applications require functionality in both conditions [38]. Additionally, following the trend of the majority of natural, biobased materials, collagen exhibits inferior electrical performance compared to conventional electronic materials. To overcome the latter limitation, dual functional bio-composites are often prepared through the combination of collagen with conductive materials [79]. The formation of gelatin occurs through the partial hydrolysis of collagen when it is treated with either an acid or an alkali. Gelatin is a protein soluble in water that presents notable properties, such as biocompatibility and biodegradability, gelling, and ion-exchange behavior, but it has inferior mechanical properties [75]. According to the literature, gelatin exhibits low thermal resistance, resulting in a reduction in the mechanical toughness at elevated temperatures. Moreover, this protein can easily dissolve in polar solvents, including water, glycerol, and acetic acid, indicating non-optimal environmental stability for printed electronic applications. Its high sensitivity to alterations of temperature, as well as humidity, can accelerate degradation, thereby undermining the final electronic device’s stability, reliability, and functional longevity. Furthermore, gelatine displays inferior electrical conductivity compared to conventional materials (e.g., silicone), a parameter that restricts the scope of its potential electronic applications. In addition to the above, gelatine has been reported to demonstrate compatibility issues with certain conventional electronic components [56].

It is worth taking into consideration that gelatin conductive hydrogels are viewed as promising materials for manufacturing flexible wearable sensors because of their unique porous structure, high capacitance, flexibility, non-toxicity, excellent biocompatibility, and biodegradability. Therefore, various reinforcement species (such as metal nanomaterials, carbon-based materials, and polymers) have been added to gelatin, leading to significantly advanced gelatin conductive hydrogels [80]. Landi et al. developed an eco-friendly temperature sensor utilizing an affordable gelatin–graphene nanocomposite material. The gelatin serves as a binder to unite the hydrophobic graphene filler with the water–glycerol molecules and functions as a solid electrolyte, acting as a proton conductor [81].

#### 3.2.4. PLA-Based Nanocomposites

PLA is one of the most prevalent biopolymers and has numerous applications in the packaging, medical, and pharmaceutical fields. However, the research on its use for optics and electronics is limited [75].

Santos et al. developed a flexible, biodegradable, and metal-free 3D-printed thermal sensor by coating a PLA filament with PLA/CNT-based ink. The results showed that the PLA/CNT-based sensor can operate as a thermoresistor and thermoelectric [82].

### 3.3. Polymer Blends and Their Application in Electronics

Polymer blends derive from the combination of at least two polymeric materials [83]. The final blends exhibit properties that lie between those of the individual component polymers [43], however, in most of the cases, the adjustment and tailoring of their chemical, physical, mechanical, and other properties is successfully achieved; therefore, blends with enhanced characteristics are generated [43,83]. Consequently, the blending of widely available polymers serves as a facile and cost-effective method of producing novel, improved materials [84]. Blends are commonly employed in multiple domains, and PE are a notable example.

The production of polymer blends for electronic applications has progressed beyond conventional materials, with various studies reporting the incorporation of biobased polymers. Particularly natural polymers, such as starch, are frequently blended with synthetic polymers in order to achieve improvement of their mechanical properties and alignment with the requirements of multiple practical applications [85]. Chemical properties are additionally optimized through blending. For instance, the elevated hydrophobicity of methylcellulose presents a considerable limitation for the polymer’s application as a potential substrate for flexible bioelectronic devices [86]. Blending methylcellulose with starch consists of an effective approach to overcome this issue.

**Figure 6:**
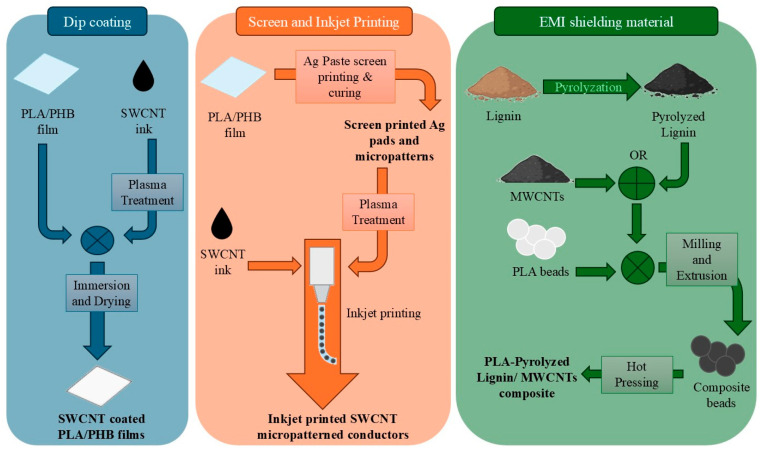
Schematic representation of the processes employed for the fabrication of PLA/PHB-based materials and electrical components [94].

Chitosan, cellulose, and cellulose derivatives have been widely investigated in blends for electronics [87,88]. In an earlier study by Basavaraja et al., carboxymethyl cellulose’s (CMC) blend with polyaniline exhibited improved electrical conductivity [89], a result that was similarly observed by Alonso et al., when bacterial cellulose was combined with the same polymeric material [90]. In a recent article by Kumar et al., chitosan/poly(vinylpyrrolidone) (PVP) blends were employed as flexible substrates for gas-sensing and semiconducting devices [91,92]. Furthermore, biodegradable substrates for flexible and wearable electronics in optoelectronic devices have been formed through the blending of chitosan with potato starch [92,93]. Additional biobased polymers that have been utilized in blends for electronic applications are PHAs, PLA, lignin, and collagen [73]. Blends incorporating biobased polymers have also been examined for applications specifically in PE. In a recent work, Bozó et al. synthesized PLA/PHB blends and examined them through processes commonly employed for the production of electronic components, such as inkjet and screen printing [94]. The blends were further reinforced with pyrolyzed lignin and multiwalled carbon nanotubes. Compared to the neat blends, the doped ones exhibited exceptional electromagnetic interference shielding properties at frequencies between 18.0 and 26.5 GHz. Figure 6 presents an overview of the processes applied for the preparation of PLA/PHB-based materials and electrical components.

**Figure 7:**
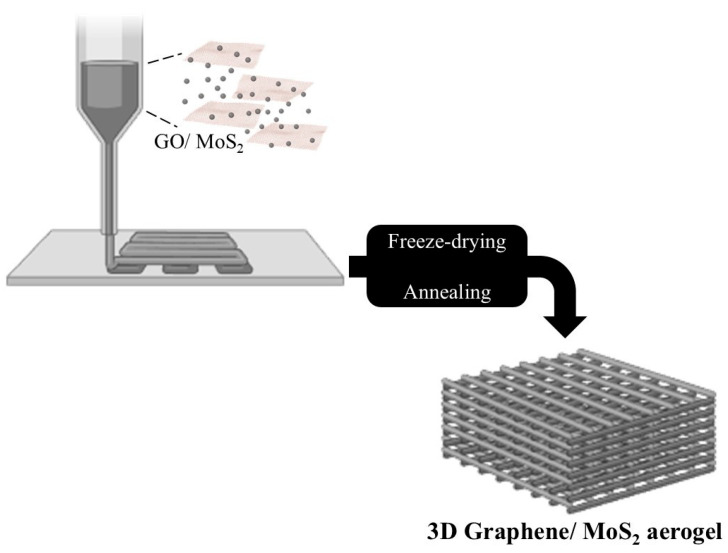
Illustration of the direct ink writing process of graphene oxide (GO) and MoS_2_ aerogel electrodes [98].

Massey et al. utilized PVA/carrageenan (CAR) and PVA/PCL blends for the formation of bilayer dielectric stacks [95]. The PVA/CAR blend was employed for the formation of metal-insulator-metal (MIM) capacitors and organic thin-film transistors (OTFTs), while the PVA/PCL blend was utilized solely to produce OTFTs. The addition of biobased polysaccharides, such as carrageenan, in PVA contributed to the enhancement of the latter’s mechanical properties. Cross-linking of the two polymers occurred through a freeze–thaw process. Moreover, the dielectric PCL when blended with PVA elevated the structural integrity of the final OTFT. Both PVA/CAR and PVA/PCL blends displayed reasonable potential for applications in sustainable direct PE. Furthermore, Tosto et al. blended a biobased epoxy resin with a commercial daylight-curable acrylate resin, for the formation of 3D-printed components through liquid crystal display (LCD) printing [96]. The resins were tested in various mass ratios, aiming for the improvement of their thermomechanical behavior. LCD 3D-printed components demonstrated potential for integration in printed circuit board standoffs. Additionally, Plamieri et al. synthesized ethyl cellulose (EC)/hydroxypropyl cellulose (HPC) blends of various ratios to facilitate the tuning of the obtained materials’ properties (chemical, physical, and mechanical) [97]. Consequently, the produced substrates demonstrated compatibility with a substantial number of processing techniques, including screen printing. Moreover, Chandrasekaran et al. generated 3D-printed aerogels by blending hydroxypropyl methyl cellulose with the copolymer Pluronic F-127 [11,98]. Inside the blend, graphene was additionally incorporated. The final aerogels were constructed through direct ink writing (DIW) printing (Figure 7), with the intention of being applied as electrodes for water splitting. In a recent article, Luoma et al. examined a stereocomplex PLA blend as an alternative substrate for flexible and hybrid electronics [99].

To conclude, biobased polymer blends’ utilization in electronic applications—specifically in PE—has increasingly been documented in scientific research (Table 1). However, it is important to underline that according to the literature, plain or undoped polymer blends are not widely used in electronic applications. Instead, research has focused on composite materials based on polymeric blends, which offer enhanced functional properties suitable for electronic device performance.

### 3.4. Substrates

Substrates are the solid supporting materials of electronic devices’ components, such as dielectric and semiconducting layers and electrodes [5,38,103]. Currently, one can attribute substrates’ broad employment to their suitable physical properties like strength, flexibility, and surface energy for the successful deposition and effective adherence of the residual electronic elements [104,105]. Furthermore, in certain cases they provide protection, for example, against moisture and oxidative degradation [106]. Especially for flexible electronics, the substrate is acknowledged as the most vital element considering that it determines the flexibility of the entire device [107]. Significant factors for the selection of the substrate material include its thermal [108] and dimensional stability, the solvent resistance it demonstrates [59], its compliance with the processing stages during the device’s fabrication, and the mechanical robustness it exhibits [108]. Insulating properties are also frequently desired [5,38]. In addition, specifically stretchable electronics, as well as the majority of PE, require the substrate to demonstrate flexibility [38].

Τhe employment of biobased polymeric materials as substrates specifically in the field of PE has been addressed in numerous studies, highlighting their potential. The requirements for the substrate, and consequently the utilized biobased materials, are determined by the final application and the selected printing method. These requirements include factors such as the substrate’s thickness, its wetting properties, its inherent chemical stability toward solvents and additives, the dimensional stability it exhibits in increased temperatures, as well as under UV-light or during plasma treatment, and, also, the substrate’s surface energy [105]. Examples of biobased polymeric materials applicable as substrates in PE include cellulose, PLA [4,43,73,109], silk, collagen, gelatin [4], poly(lactic-co-glycolic acid) (PLGA) [4,110], and starch [85,102].

In the following sections, we present and critically discuss the scientific progress reported in recent years concerning the use of biobased polymers, including cellulose and its derivatives, PLA, silk, and other polymeric materials, as substrates in PE. The most commonly employed polymeric materials for substrate formation are poly(ethylene terephthalate) (PET), poly(ethylene naphthalate) (PEN), poly(ether-ether-ketone) (PEEK), poly(dimethylsiloxane) (PDMS) [22], polyimides (PI), poly(ether sulfones) (PES), poly(ether imides) (PEI) [59], poly(vinylidene fluoride) (PVDF), polyurethane (PU), and thermoplastic polyurethanes (TPU) [5].

**Figure 8:**
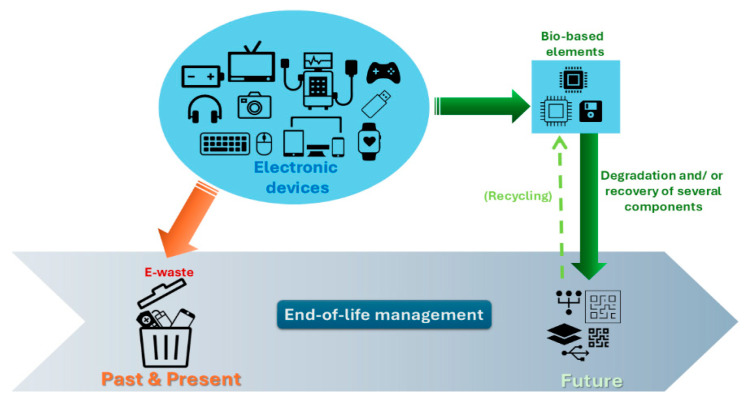
Schematic illustration of the past, present, and optimal future end-of-life (EoL) management of electronic devices.

Considering that the substrate’s width and thickness is substantial compared to the residual elements, the device’s degradation behavior is strongly influenced by the substrate’s material decomposition rates [38,103,108]. Consequently, the utilization of the aforementioned fossil-based polymers induces slower degradation and notable accumulation of electronic waste [38,85], while also augmenting reliance on petrochemical resources. Aiming to restrain these undesirable repercussions, the scientists’ interest is currently shifted to the employment of biobased polymers as raw starting materials for the synthesis of electronic devices’ substrates (Figure 8) [108].

Several polymeric materials deriving from biomass and other natural sources (microorganisms, algae, agricultural waste, etc. [43]) are biodegradable and/or recyclable, however, this is not universally the case (for example, biobased polyethylene is not biodegradable). Nevertheless, their use remains beneficial, compared to conventional materials, for reducing dependence on fossil resources [111].

#### 3.4.1. Substrates from Cellulose and Its Derivatives

Cellulose represents the most abundant [43], broadly acknowledged, and financially viable biobased polymer [108]. According to the literature, cellulose has been utilized for the synthesis of substrates that exhibit dielectric properties [73], for printed circuit boards’ (PCB) substrates [4,43], as well as for more specific electronics’ components, such as substrates of inkjet-printed electrodes for novel organic diodes [112]. The various employed cellulose derivatives and forms were cellulose acetate, wood-derived cellulose, cellulose–laponite composites [4], cellulose nanofibers [4,43], and cellulose nanopapers [112].

**Figure 9:**
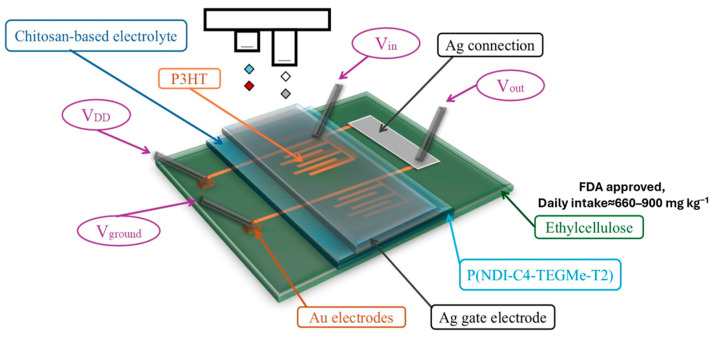
Illustration of the ethylcellulose-based device with chitosan-gated transistors (P3HT: poly(3-hexylthiophene), and P(NDI-C4-TEGMe-T2): a modified version of P(NDI2OD-T2) bearing polar triethylene glycol-based (TEG-based) side chains), employed as an electron transporting material in n-type devices [114].

Regarding recent work progress, focusing on flexible hybrid electronics, Luoma et al. employed cellulose acetate propionate and regenerated cellulose film, known as NatureFlex^TM^, as substrate materials [59]. The thermal and surface properties of the biobased substrates were further enhanced through annealing and film orientation. Both materials exhibited transparency similar to conventional substrates and printed pattern resistance values better than those of commercial and commonly employed PET films. The selected printed method was screen printing and the printability of silver ink on these cellulose-based substrates was suitable, leading to the conclusion that such biobased materials are promising for flexible and biodegradable electronics. The same scientific group additionally studied the consequences of accelerated aging on the properties of the aforementioned materials [113]. They concluded that none of the substrates’ properties were altered solely in elevated temperatures, as well as in elevated temperatures and humidity. Furthermore, it was reported that under UV-A radiation exposure, limited to no changes were observed for NatureFlex^TM^, and when subjected to thermal cycles ranging from −45 °C to 65 °C, both materials demonstrated stability. In another article, nanocellulose papers (NCPs) were employed as substrates for green printed electronic applications. In total, three printing techniques were tested: inkjet printing, screen printing, and direct ink writing. Despite the fact that decreased sheet resistance values were obtained, the flatness of the nanocellulose paper substrates was not optimal. Furthermore, bending tests confirmed the ability to employ these materials for flexible, printed electronic applications; however, it was reported that improvements are still necessary. Additionally, in a recent research paper, Palmieri et al. utilized an ethyl cellulose/hydroxypropyl cellulose blend for the generation of flexible electronics’ substrates [97]. The blends’ production process was facile and environmentally friendly. Their employment enabled the tuning of mechanical and chemical properties by adjusting the ratio of the individual polymers. The aforementioned materials exhibited excellent mechanical stability and elongation properties. Their process suitability was examined through UV photolithography; however, it was stated that the application of screen printing is also plausible with a proper polymer ratio. Furthermore, Jaiswal et al. studied a nanocomposite of cellulose-derived materials [3]. Cellulose nanofibril and hydroxyethyl cellulose were employed for the synthesis of a composite applicable as a substrate for printed flexible electronics. Various ratios of the two components were examined until nanocomposites with exceptional mechanical and optical properties were obtained. The selected material was utilized as a substrate for the formation of a wearable electrocardiograph patch through sheet-to-sheet printing and a manual assembly process. Nevertheless, it was reported that the fabrication of the device is also achievable through automated roll-to-roll printing. The electrocardiograph patch was tested on a human volunteer, where it successfully monitored the heart’s electrical signal. Additionally, the nanocomposite-based patch was subjected to soil and marine degradation tests, which induced the deterioration of the cellulosic substrate and facilitated the recovery of the printing ink and the electronic components. Sharova et al. performed inkjet printing of inert gold electrodes on an edible ethyl cellulose substrate [114]. The primary aim was to generate edible, low-voltage operating transistors, as depicted in Figure 9. The aforementioned devices demonstrated suitable electronic behavior, as evidenced by a high-mobility capacitance product, an adequate on–off current ratio, operational stability during atmospheric exposure, and a shelf-life extending up to one month. Moreover, due to their flexibility and reduced dimensions, these devices were reported as applicable in edible electronic carriers, such as pharmaceutical delivery vehicles.

**Figure 10:**
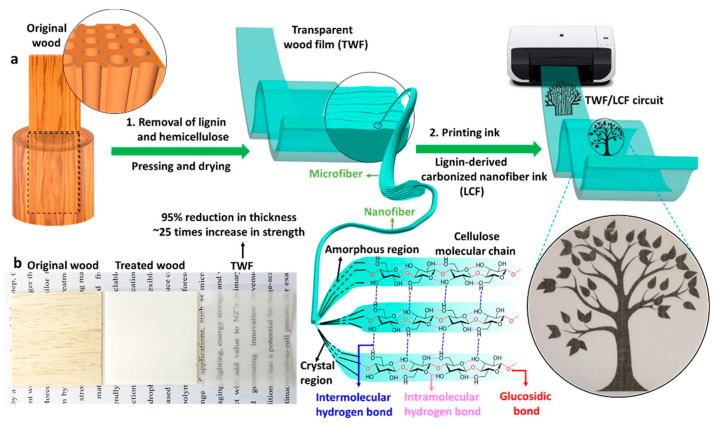
Fabrication of TWF for use in flexible electronics. (**a**) Schematic of the process: Lignin and approximately half of the hemicellulose are extracted from wood tissue, followed by pressing and ambient drying of the treated material. Hydrogen bonding links the collapsed cell walls. TWF’s hierarchical architecture features cellulose microfiber bundles, nanofibrils, and chains with both crystalline and amorphous domains. A biobased LCF ink is inkjet-printed as a tree-shaped circuit onto the TWF substrate. (**b**) Photos showing untreated wood, processed wood, and final TWF [117].

Additionally, Morfa et al. utilized cellulose diacetate flattened foils as substrates for the development of organic light-emitting diodes (OLEDs) [115]. The devices were fabricated through a combination of processes, including inkjet printing. The substrate’s performance was optimal and described as comparable to that of conventionally employed materials. Furthermore, in a very recent article by Wang et al., methylcellulose along with tannic acid were applied for the synthesis of substrates employed in flexible printed circuit boards (FPCBs) [116]. The obtained substrate material was a reversibly cross-linked hydroplastic, which exhibited thermal and water stability, high storage modulus, and increased mechanical strength even after 15 days underwater. The produced FPCBs were destined for sensing applications; therefore, sensing components were 3D-printed on the substrates. It was stated that the hydroplastic could undergo several recycling cycles and was also able to dissolve in medical alcohol, therefore enabling the recovery of the 3D-printed electronic components. The sensing devices’ performance in terms of sensibility, reliability, and durability, even after immersion in water, was noteworthy. Lastly, in a different article by Fu et al., wood-based cellulose was employed for the synthesis of substrates for flexible electronic circuits [117]. Starting from wood, lignin and hemicellulose were eliminated, while the cellulose nanofibers were preserved with their original alignment. For the printing process, a conventional printer was employed, and lignin-derived carbon nanofibers were utilized as the conductive ink (Figure 10). The substrate material exhibited flexibility simultaneously with increased mechanical properties, like tensile strength, notable transparency, and conductivity. According to the article, these devices have potential applications in sensors and wearable devices. Table 2 summarises cellulose based substrates and their applications in the field. 

#### 3.4.2. PLA-Based Substrates

PLA is an additional extensively examined biobased polymer for the formation of PE substrates. It has been investigated as a substrate material for PCBs, all-inkjet-printed organic electrochemical transistors [4,43], bioelectronic applications [43], biodegradable sensors [73,118], as well as wireless sensor networks and frequency sensing applications [118].

In their recent work, Nassajfar et al. employed PLA as the substrate material for screen-printed PCBs [109]. The compatible inks were silver-, copper-, and graphite-based, while the reported final application of the devices was potentially in food packaging sensors. PLA’s annealing and orientation have been suggested as successful techniques for the enhancement of its properties when employed as a substrate material for flexible PE [99]. In more details, various forms of PLA were examined, including standard PLA, high-heat PLA (hhPLA), and stereocomplex PLA blends (scPLA). Machine direction, biaxial orientation, and annealing improved the performance of all examined PLA forms, which were further studied as substrates of LED foils. Two printing techniques were applied for the formation of the electronic structures: a pilot-scale R2R rotary printing process and a lab-scale S2S screen-printing process. The produced systems displayed dimensional and electrical properties that were comparable to those of conventional counterparts fabricated on PET, despite the fact that decreased temperatures were applied.

**Figure 11:**
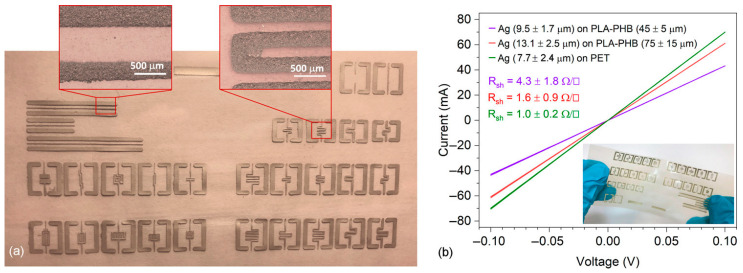
(**a**) Illustration of the screen-printed silver micropatterns on the surface of the PLA/PHB blends and (**b**) current–voltage characteristics of inkjet-printed silver lines on PLA/PHB substrates of two thicknesses, compared to a PET reference substrate [94].

Luoma et al. incorporated the utilization of oriented and annealed PLA as a substrate for general printed hybrid electronics [59]. For the printed components silver ink was employed, and the selected technique was screen printing. The substrate material demonstrated increased optical transmission compared to conventionally employed PET, and it was, therefore, proposed for applications where optical transparency, as well as stiffness and tensile strength, are required. The authors of the aforementioned article additionally examined the consequences of accelerated aging on the properties of the PLA substrates [113]. They concluded that increased temperature, as well as simultaneously elevated temperature and humidity, do not affect the substrates, while UV-A radiation exposure generates limited to no alterations. Following their exposure to diverse environmental conditions, PLA substrates sustained elevated light transmission values and desirable visual characteristics. Hakola et al. constructed smart labels utilizing PLA as the substrate material [119]. PLA blends with poly(hydroxybutyrate) (PHB) were utilized by Bozó et al. as flexible dielectric substrates for printed conductors [94]. The blends were initially plasma-treated, and the applied printing methods were inkjet and screen printing for the formation of carbon nanotube and silver patterns, respectively. The screen-printed silver nanopatterns are depicted on Figure 11. Inkjet printing, according to the article, represents a potential method for the generation of micropatterns on biobased plastic materials that exhibit properties similar to those observed when conventional substrates are employed.

**Figure 12:**
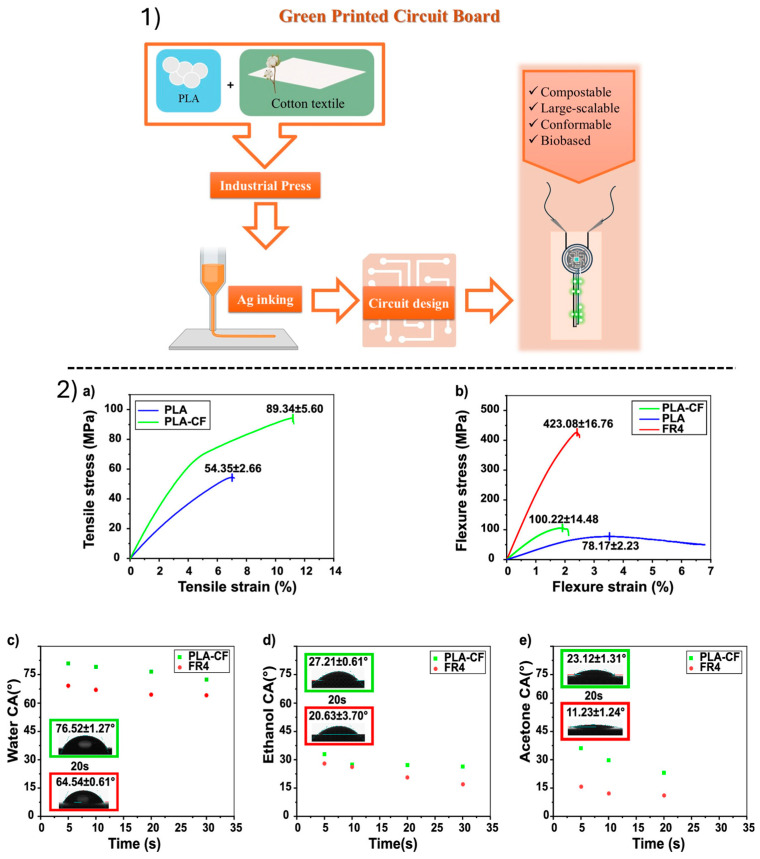
(**1**) Illustration of the fabrication process of green PCBs utilizing a PLA–cotton fabric fusion as a substrate material and (**2**) (**a**) tensile stress–strain curves for neat PLA and PLA reinforced with cotton fabric (PLA-CF). (**b**) Flexural bending tests on neat PLA, FR4, and PLA-CF. (**c**–**e**) Water, ethanol, and acetone contact angles for PLA-CF compared to FR4 as a reference material [122].

Gomes et al. employed PLA as a flexible and sustainable substrate of electronic devices with sensing abilities [120]. The devices’ fabrication method was screen printing, and the final products were described as stable, robust, reproducible, and facile to employ, while they also exhibited sufficient sensitivity to certain biomarkers. Additionally, the substrates’ mechanical strength toward substantial strains was also adequate. The aforementioned flexible sensing device was successfully coupled with a Sensit BT portable analyzer for the detection of biomarkers in undiluted human urine samples. Moreover, the analyzer could establish a Bluetooth connection with smartphones, tablets, or laptops. PLA substrates were also utilized by Goument et al. for in-mold electronics, the fabrication of which includes screen printing of conductive ink [121]. The ink exhibited sufficient adhesion with the substrate; however, improvements are still necessary, especially regarding PLA-based substrates’ thermal behavior. Honarbari et al. investigated the use of PLA and cotton fabric layers as PCB substrates (Figure 12) [122]. The PLA layers were infused inside the weave of the cotton fabric through hot pressing. Interaction tests with water, ethanol, and acetone were performed on the PLA–cotton fabric substrates, which showcased promising results. Therefore, the printing of conductive inks based on the previously mentioned solvents was reported as plausible.

Additionally, Välimäki et al. employed PLA as a substrate in ultra-thin organic photovoltaics (OPVs), which incorporated gravure and screen-printed materials, such as PEDOT:PSS, carbon, and amino acid/heterocycles [6]. Within the same study, PLA was further examined as a substrate for printed and hybrid integrated LED foils. From the results obtained it was concluded that PLA represents a potential alternative substrate for the fabrication of OPVs, as well as for the development of printed and hybrid integrated electronics through scalable manufacturing methods. Finally, PCB substrates from flax-reinforced PLA were also recently reported by Géczy et al. [123]. The substrate was additionally laminated with copper, and despite the adequate shear strength of the printed components, multiple improvements of the material’s properties were still required.

#### 3.4.3. Silk-Based Substrates

Another biobased polymer applicable as a substrate in PE is silk. Silk has been reported in split-ring resonators with metamaterial arrays, fabricated through water-based micro-contact printing for bioelectric and bio-photonic applications, such as bio-tracking, bio-mimicking, and implantable biosensors [118]. Composite materials based on silk have also been employed. A representative example is titanate nanosheets–silk nanocomposites, which were restructured into various forms through fabrication processes, such as inkjet printing [75].

In recent literature, Wang et al. blended silk fibroin with certain additional materials and utilized the final blend as the substrate for the formation of self-healable electronic tattoos (E-tattoos) with sensing properties [124,125]. The final substrate material was a graphene/silk/fibroin/Ca^2+^ combination, and the fabrication of the E-tattoos was achieved through the printing techniques of screen printing and direct writing. Due to the incorporated graphene, the aforementioned tattoos were able to respond to various environmental stimuli, including strain, humidity, and temperature. Furthermore, after a damage test with water, the E-tattoos were fully healed in just 0.3 s. They were additionally applied as sensors for the detection of electrocardiogram, breathing, and temperature signals. The self-healing properties, enhanced sensitivity, rapid response, and long-term stability of the E-tattoos rendered them highly suitable for epidermal electronic applications.

#### 3.4.4. Substrates from Other Biobased Polymers

In addition to cellulose, PLA, and silk, several other biobased polymers have been investigated as substrates in PE, including polylactic-co-glycolic acid (PLGA) [110], which is applicable in implantable and bioresorbable devices, and starch [7]. The latter is an abundant, inexpensive natural polysaccharide that forms soft and transparent substrates suitable for flexible electronics [85] and printed bioelectronic devices [102].

**Figure 13:**
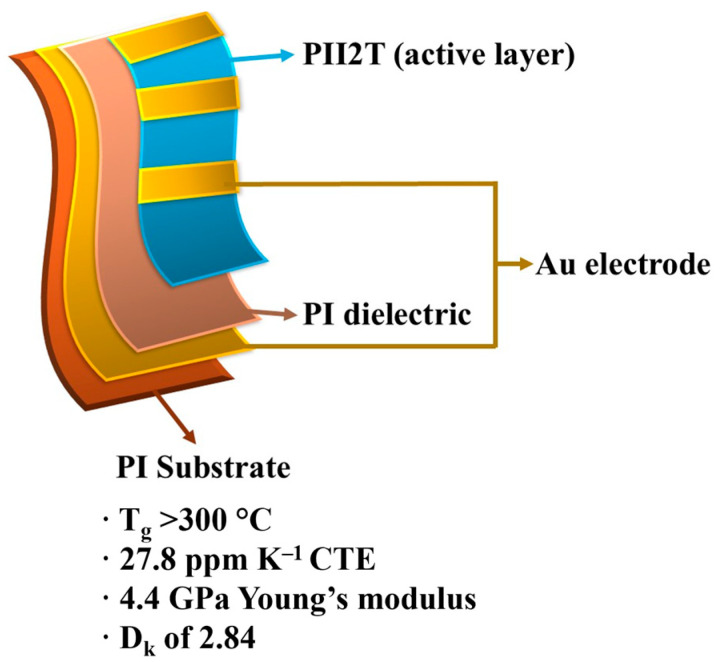
Detailed illustration of the OFET device and its individual components. Graphs of the field-effect transistor’s transfer characteristics [128].

Furthermore, in a recent article, Hwang et al. employed pollen-based materials as biopolymer substrates of flexible and stretchable electronics [126]. In more details, through appropriate pollen processing, sporopollenin particles were formed and applied as starting materials for the formation of stimuli-responsive, flat-surfaced, and complex structures. Adjustments of sporopollenin substrates’ chemical, mechanical, and thermal properties aimed at their extended and reliable utilization in conventional electronics. Fabrication processes, such as direct ink writing and transfer printing, were feasible on the aforementioned substrates for the formation of stretchable physiological sensors, heaters, and generally wearable biomedical devices. In a different article by Hao et al., gelatin–alginate hybrid hydrogels with stencil-printed liquid metal were employed as substrates of soft electronics with sensing properties [127]. The hybrid hydrogels showed self-healing properties and the fabricated devices could sense different stimulus, such as strain, temperature, heart rate, and pH. The strain sensing element of the devices could successfully detect the human arterial pulse. Moreover, as a result of their thermally induced sol-gel transition, the hybrid hydrogels could achieve seamless contact with objects possessing complex geometrical configurations. The devices were reported to be biodegradable and recyclable, therefore enhancing the general idea of sustainable electronics and aiming toward the reduction of electronic waste. Lastly, Hung et al. reported the incorporation of biomass-based polyimides as substrates for flexible organic field-effect transistors (OFETs; Figure 13) [128]. The polymeric materials derived from (3R,6S)-hexahydrofuro[3,2-b]furan-3,6-diyl bis(1,3-dioxo-1,3-dihydroisobenzofuran-5-carboxylate), also known as ISBESA, the synthesis of which can be achieved utilizing isosorbide bioresources as starting material. The polyimides were additionally examined as interlayer dielectric materials. OFETs’ fabrication process included the transfer printing of the active layer onto the polyimide-based structure. The produced devices exhibited adequate electrical performance, comparable to their conventional silicon-based counterparts. Moreover, they showcased stability during their operation, even after thermal treatment at 150 °C or 1000 bending cycles. Consequently, the biobased polyimide substrates were described as promising for future electronic applications.

In conclusion, as part of the scientific community’s effort to reduce dependency on fossil resources, minimize electronic waste, and facilitate the development of sustainable electronics, multiple biobased polymers have been examined as substrates for printed electronic applications. The most extensively reported are cellulose, cellulose derivatives, and PLA. Apart from pure polymers, blends and composites are also frequently applied. Electronic devices, such as PCBs, sensors, biosensors, and flexible electronics, constitute the most prevalent applications of biobased polymer substrates. Despite their promising performance in most instances, it is widely acknowledged in the literature that further improvement of those materials is necessary in order to scale up toward commercial production.

### 3.5. Biobased Conductors and Semiconductors

In the past years, scientists considered that all types of polymers present an electrical insulator’s performance. The invention of polyacetylene and its conducting properties led to the invention of other polymers with similar electrical performance, the conductive (or conducting) polymers (CPs), meaning organic materials that can conduct electricity. Conductors and semiconductors are considered the total of polymers, fillers, and additives that exhibit high values of electrical conductivity, resulting in their utilization in PE for many of them. Such conductive polymers are polypyrrole (PPy), polyaniline (PANI), poly(3,4-ethylenedioxythiophene) polysulfonate (PEDOT:PSS), and poly(p-phenylene vinylene) (PPV), which present a semiconductor’s behavior. Aiming to increase the conductivity of both these and mainly all other polymers that are insulators, a plethora of nanofillers can be incorporated onto polymer matrices. The most popular ones are graphene and its derivatives, carbon nanotubes (CNTs), graphite and its derivatives, as well as metal nanoparticles [38,129,130].

However, most of these polymers and nanofillers are included in fossil-based products and they are obtained by synthetic methods, such as chemical oxidation, electrochemical polymerization, electrospinning, and template-assisted techniques, resulting in being considered non-biobased. Polythiophenes like PEDOT:PSS are an exception and are included in biobased polymers [38]. For other nanofillers, due to their small size, it is possible to enter the human body. For instance, CNTs are derived mainly from ethanol and methane, which are petroleum-based products [131]. The slow degradation rate of CNTs, the minute dimension, and high surface area cause important environmental concerns and dangers about human health when organisms are exposed or inhaled [132]. Respectively, metal nanoparticles released in the form of metal ions to human organisms may cause serious health issues and dysfunctions in many systems of organisms. For instance, the release of nickel ions possibly leads to DNA damage and cancer. Zinc oxide NPs, with wide interest in PE, can insert into organisms by respiratory, digestive tracts, dermal, and parenteral paths [133]. In addition, silver NPs can enter the organism, when it comes into contact with the human skin, causing cellular damage and cytotoxicity [134]. Consequently, we observe that the toxicity of conductive nanofillers and human problems that cause them constitutes a serious limitation for their use.

While the environmental issues constantly make their appearance, it is easy to see that the value of biomass waste is high and necessary to be utilized in many application fields [135]. Therefore, in the PE sector, the replacement of traditional conducting fillers from biobased conducting additives from renewable feedstocks or the conversion of non-biobased ones to biobased is possible to lead to a more sustainable situation. In the following paragraphs, the main additives that can act directly as a conductive additive or a precursor to form conductive carbon materials are presented. These are lignin, cellulose, starch, biochar, and bio-graphite. Polythiophenes are also described, as a biobased group of conductive polymers.

#### 3.5.1. Lignin-Based Conductors

Lignin is an aromatic biopolymer derived from wood and vascular plants. It is considered the most abundant aromatic biopolymer in nature and one of the most abundant generally. Lignin exhibits excellent biocompatibility, cost-effectiveness, and environmental friendliness, while a plethora of functional groups are contained in its structure, making it suitable to utilize it as a biobased filler in polymer matrices [136]. In the context of the use of biobased components (polymers and additives), lignin constitutes an ideal biobased precursor for its conversion to small carbonaceous compounds with enhanced electrical properties, such as CNTs, while simultaneously a high-value utilization can be achieved [137]. This process is, usually, called carbonization, and it includes four main types: pyrolysis, hydrothermal, thermal plasma, and laser-abated direct carbonization [138]. Generally, in carbonization processes, lignin is subjected to high-temperature (500 °C) breaking of alkane bonds and to release of gases, such as CO_2_ and CH_4_, and removal of functional groups [137]. The forms of lignin-derived carbon materials can be fibers, foams, aerogel, and activated and porous carbon [139]. The huge number of functional groups of lignin facilitates the further modification of received products and combination with other additives. Additionally, the selection of the desirable carbon additive for each application is possible, because features such as the porosity and microstructure of lignin-derived carbon additives are affected by the types of their precursors [140].

In the literature, several works have focused on the preparation of lignin-based carbon additives with enhanced electrical conductivity for many possible applications, such as supercapacitors, lithium-ion batteries, and wearable sensors. Tu et al. synthesized lignin-based CNTs using industrial lignin derived from enzymatic hydrolysis [137]. Respectively, Du et al. used successful lignin-based CNTs and cellulose nanofibers in order to prepare a 3D-printed extruded aerogel for application as wearable sensors [141]. Yang et al. prepared lignin-derived graphene with high porosity for application to wearable sensors and they reported an advanced stability and sensing ability, suitable for using as a strain sensor [142]. In another work, Culebras et al. synthesized biobased carbon fibers, aiming to apply them to lithium-ion batteries, using PLA/lignin and TPU/lignin composites as biobased precursors [143].

In Table 3, fossil-based and lignin-based graphene and CNTs are compared, in terms of electrical conductivity, tensile strength, and thermal conductivity. According to this quantitative comparison, it is only fair to conclude that it is possible to prepare biobased graphene and CNTs with analogous electrical conductivity, using lignin as a precursor. Lignin-based graphene and CNTs can effectively replace fossil-based ones for applications that require high electrical conductivity, including printed electronics.

#### 3.5.2. Cellulose-Based Conductors

Cellulose is a biopolymer received mainly from photosynthesis of higher plants and is synthesized by organisms such as bacteria, animals, and algae. Gram-negative bacteria can synthesize nanocellulose. Bacterial cellulose also has been used to produce carbon materials with 3D porous structures. The presence of -OH groups makes it hydrophilic and facilitates possible chemical modifications. As the most abundant biopolymer in nature, cellulose has been extensively utilized for the production of biobased carbon additives via carbonization methods. Their formed structure depends on the type of precursor, the carbonization temperature, the carbonization process, and the heating speed, and they can be microfibers, microspheres, nanosheets, nanoshells, nanocrystals, and nanofibers. However, in order to apply them as electrodes for batteries, the carbonization process does not significantly increase the electrical conductivity of materials due to their changes in crystalline microstructures, which negatively affects the charge transport. Aiming to overcome this problem, two possible approaches are the combination of cellulose-derived carbon materials with another one with high conductivity or the incorporation of functional groups that facilitate the charge transport. In the context of sustainability, the desirable method and material are selected [152,153]. The potential applications of cellulose-derived carbon additives in PE are presented in many publications in the literature. Omoriyekomwan et al. utilized cellulose for the production of biobased CNTs via the microwave pyrolysis for energy storage devices [154]. Yaqoob et al. produced biobased graphene from cellulose in order to fabricate fuel cells [155]. Additionally, Tang et al. prepared an array of cellulose-based CNTs doped with Fe and N in order to fabricate fuel cells [156].

#### 3.5.3. Starch-Based Conductors

Starch constitutes the second most abundant polysaccharide in nature and an easily obtained biopolymer. The environmental challenges caused by the use of petroleum-based polymers and additives have turned the scientific interest into the utilization of starch not only as a biobased polymer but also as a precursor to produce biobased carbon additives, especially hard carbon [157]. It has a spherical morphology, high porosity, and tunable structure but its low thermal stability leads to the release of several volatile gases during pyrolysis, resulting in the loss of spherical morphology. For this reason, modifications and activations in starch’s structure are considered necessary for the extension of electronic applications of these additives [158]. For instance, Hu et al. received corn-starch-derived carbon materials using ZnCl_2_ as an activating agent to use them for manufacturing supercapacitors. According to their results, they reported enhanced electrochemical properties of samples and a potential application not only as supercapacitors but also as mesoporous materials [159]. Kim et al. prepared a starch-derived carbon material in order to use it for the fabrication of rechargeable seawater batteries [160]. In addition, Liu et al. prepared carbon dots derived from several sources of starch with applications to printing technology [161].

#### 3.5.4. Biochar-Based Conductors

**Figure 14:**
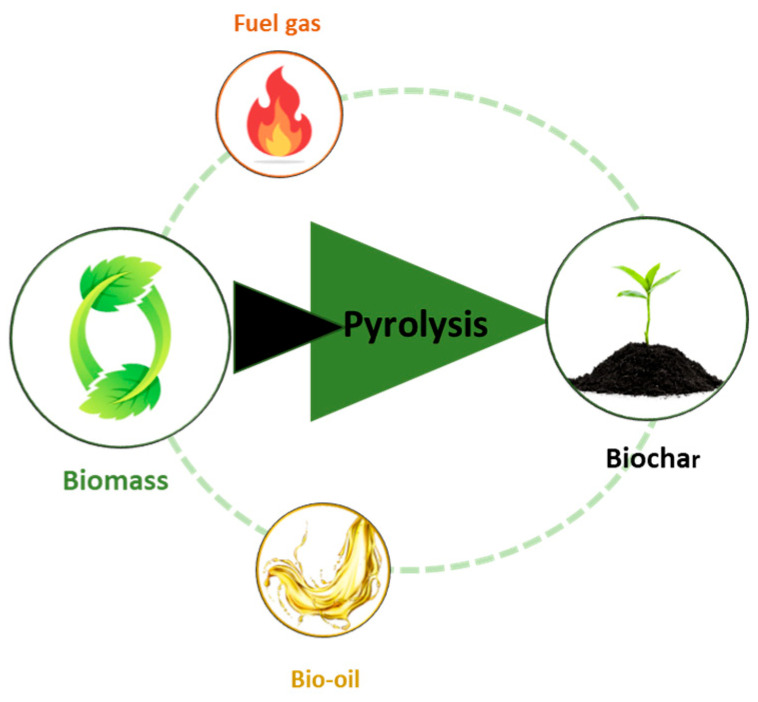
Production processes of biochar [164].

Biochar constitutes a solid biobased carbonaceous material derived from the pyrolysis of renewable sources, such as coconut, rice husks, and walnut shells, and absence of oxygen. It consists of a plethora of aromatic rings and condensed structures, a feature that differentiates it from other organic matter [162]. The type of raw material and the production conditions primarily determine the physical and chemical properties of biochar. Generally, it presents enhanced electrical conductivity, mechanical flexibility, biocompatibility, and high porosity. Additionally, biochar is characterized by its high recycling ability, as well as its low production, market, and energy costs, making it suitable for several application fields. Aiming to investigate it and extend the application range, biochar (Figure 14) is often subjected to modifications by the addition of activating agents [163].

In recent years, biochar has seen continuous growth in PE due to desirable electrical conductivity and environmental friendliness, as a biobased additive in several biodegradable polymers. The high carbon content and its graphitic properties lead to electron transport, explaining the high value of electrical conductivity. Especially, it is used for fabrication of energy storage devices, supercapacitors, and screen-printed electrodes, while its high flexibility is utilized for flexible and wearable electronics [165,166]. For instance, Rocha et al. prepared PLA/biochar filaments using castor oil as a biobased plasticizer in order to fabricate biosensors of metal and organic compounds. They used an environmentally friendly process and concluded that materials constitute a sustainable suggestion for the 3D printing technology [167]. Respectively, Gkiliopoulos et al. used biochar derived from tomato waste as a filler to the PLA matrix for 3D printing and they reported that composites have various potential applications, including in electronic devices [168]. In another work, George et al. fabricated a 3D-printed PLA/PBAT composite, adding coconut-derived biochar, aiming to reinforce the electrical and mechanical properties, and the results showed a positive effect on the electrical conductivity of materials [169].

#### 3.5.5. Bio-Graphite in Conductors

Graphite is derived mainly from fossil-based resources and, in combination with the high cost, its use can possibly cause serious environmental issues and is considered economically non-profitable. In the context of sustainable future development, the utilization of renewable raw feedstocks received graphite (bio-graphite) is able to face these problems and can be applied in several fields, such as PE (Table 4). Most investigations have reported the synthesis of bio-graphite at a lab scale [170]. For instance, Sagues et al. synthesized bio-graphite using several biomass-based derivatives via an iron-catalyzed one-step synthetic approach. The results show high-quality produced bio-graphite with comparable properties to non-biobased graphite, making them suitable for application as anodes to lithium-ion batteries [171]. In another work, Shi et al. used a two-step synthetic process, which included a fast pyrolysis, followed by catalytic graphitization in order to receive high-quality and high-purity (99.8%) bio-graphite from wood sawdust as feedstock [172]. In addition, Gezagehn et al. prepared nanolayered graphite derived from bamboo and pine plants, and they reported that the samples exhibited desirable thermal and electrical properties for application in supercapacitors and electrodes [173].

#### 3.5.6. Polythiophenes in Biobased Conductors

**Figure 15:**
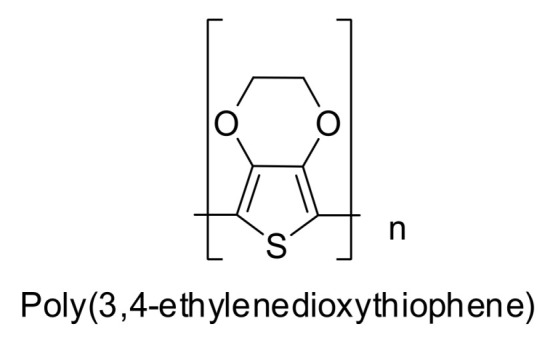
Chemical structure of PEDOT.

Polythiophenes are a group of conductive polymers that are extensively investigated for their thermal and environmental stability, as well as their optical properties. In this category are included χpolythiophene and its derivatives, like PEDOT and P3HT. Poly(3,4-ethylenedioxythiophene) (PEDOT; Figure 15) is considered the most popular one, due to its excellent electrical, optical, and mechanical features. The insolubility in water is the main drawback of PEDOT and, aiming to overcome it, a polyelectrolyte such as polysulfonates (PSS) is incorporated into the polymer matrix (PEDOT:PSS). Poly(3-hexythiophene) (P3HT) is another studied derivative of polythiophene. It is characterized by low cost, high availability, and easy processability. Yamamoto and Lin-Dudek’s synthetic methods were the first ones that were applied for the synthesis of polythiophene (PTH), while direct sol-gel, organometallic coupling reaction, and oxidative paths have also been used for its production. PEDOT:PSS and P3HT are prepared by green synthetic routes, such as synthesis in microfluids systems and electropolymerization [129].

PEDOT:PSS presents a wide range of applications in the PE field, like soft robotics systems, strain sensors, biosensors, as well as electroactive polymer and electrothermal actuators [181]. Ren et al. printed PEDOT:PSS circuits into poly(vinyl alcohol) (PVA) films, aiming to use them in transient electronics. They reported that PEDOT:PSS was printed successfully, retaining its morphology possibly due to the water solubility of PVA, as well as its electrical properties [182]. In another work, Lo et al. fabricated inkjet-printable PEDOT:PSS polyethylene oxide blends with improved electrical conductivity for applications as wearable health devices [183].

The properties summarized in Table 5 directly determine the suitability of these materials for specific electronic applications. For example, the high EMI shielding performance of chitosan-based composite materials (25–60 dB) supports their use in functional layers, where electromagnetic protection is required. Similarly, the proton conductivity of alginate (5.5 × 10^−3^ S/cm) justifies its selection as a viable electrolyte in pH or humidity sensors, where efficient ion transport is required. On the other hand, the thermal stability of scPLA and lignin-derived carbon structures allows these materials to withstand the thermal processing of conductive inks, enabling the fabrication of high-resolution conductive pathways on flexible bio-substrates. These correlations demonstrate that the transition to bioelectronics is not merely an environmental choice, but a strategic choice based on the unique functional profiles of each biopolymer.

## 4. Applications in Organic Bioelectronics and Devices

### 4.1. Organic Bioelectronics

Organic bioelectronics is an area of research that is related to π-conjugated organic electrical (semi)conductors, usually in polymer form, which are electronically linked with biological systems. Organic electronic materials are included in the abiotic element of the interface and the more biocompatibility they present, the better the interactions with the biotic environment will be [190].

Bioelectronics advanced the progress done in precision medicine and clinical diagnostics, offering bidirectional interfaces with biological systems that enable continuous, label-free monitoring alongside precise modulation of biological activity, thereby expanding their use to in vitro applications [191]. Their progress is also driven by the integration of sensors, lipid monolayers, organic electronic molecules, and polymers, whose tunable electrical, mechanical, and surface properties enable seamless and versatile biointerfaces.

More specific sensors are used in organic bioelectronics in the context of cell-based in vitro systems [192,193,194,195], hence detecting biomarkers in cell cultures there in-line or remotely [196]. Organic electronic materials, including conjugated small molecules [197] and polymers, exhibit mechanical compliance, negligible cytotoxicity, and ease of functionalization and modification of the material surface (or bulk). Organic electrochemical transistor (OECT)-based biosensors employing conjugated polymers have demonstrated detection limits in the nanomolar and, in optimized configurations, picomolar ranges in aqueous biological environments [161,165].

Lipid monolayers have been used into liquid–liquid phase-separated organic electrochemical transistors, enabling direct electronic monitoring of ion flow and membrane disruption. This can provide a robust platform for studying drug–membrane interactions and antimicrobial activity in organic bioelectronics without the need for membrane proteins [191].

Organic electronic molecules and polymers can be manufactured to obtain specific physical and chemical properties [198]. Their typically low mechanical stiffness (Young’s modulus around ~1 GPa) can be tailored through mixing with biopolymers or additives, enabling flexibility and mechanical matching with target tissues, resulting in conjugated polymer (CP)-based composites, such as conducting hydrogels, ideal for interfacing with ultrasoft biological systems [199,200,201]. Bansal et al., for instance [202], integrated flexible OLEDs and organic photodiodes into a muscle-contraction sensor, which can detect isometric and isotonic muscle contractions. The sensor’s output was subsequently utilized to manipulate a robotic arm, demonstrating the potential for using the device to manage artificial limbs. The sensor’s flexibility is a crucial advantage, enabling it to be positioned almost anywhere on the body. The device provided clear and reproducible optoelectronic signals with a sufficient signal-to-noise ratio to reliably distinguish between isometric and isotonic contractions, while maintaining stable performance under repeated mechanical deformation, highlighting a key advantage over rigid silicon-based optoelectronic platforms for wearable bioelectronic sensing [171].

The electrical conductivity of biosensors can easily be tuned through chemical doping, allowing CPs to transition from semiconducting to metal-like conductive states, enabling precise control of electronic performance for diverse in vitro bioelectronic applications. Depending on the polymer backbone and dopant chemistry, electrical conductivities in conjugated polymer-based bioelectronic materials have been reported in the range of 10^1^–10^2^ S m^−1^ [161,172,173,174].

It is also important to mention that in order to use organic materials in bioelectronics, surface alteration or conjugation with other biomolecules is frequently a prerequisite [203]. Their surface functionalization can be tailored through adsorption, covalent bonding, entrapment, or doping to reduce fouling, enhance cell adhesion, and improve properties, such as wettability and electroactivity, making CPs more effective for bioelectronic interfaces [204,205,206,207,208,209,210].

Current applications of organic bioelectronics include the creation of biosensing systems, neural interfaces, and tissue scaffolds, in order to regulate and stimulate cell activity. Recent bioelectronic platforms based on conducting polymer hydrogels and granular architectures demonstrate cytocompatibility exceeding 95% cell viability and stable electrical performance [161,162].

It is vital to mention the fact that organic bioelectronic materials and devices play a key role in bioelectronic medicine, providing the opportunity of developing therapies based on electrical stimulation without drugs. The implementation in regenerative medicine is also notable, as the materials that transfer electronic and ionic signals could assist self-cleaning [211].

#### 4.1.1. Biosensing

Platforms based on conducting polymers have been widely utilized for developing biosensing devices since they provide an ideal environment for the immobilization of recognition elements and function as physicochemical transducers that convert chemical signals into measurable electrical responses [212].

Organic materials exhibit various electronic properties, such as tunability, conductivity, and semiconducting properties, that contribute to their remarkable sensing abilities. Organic electronic devices like organic field-effect transistors (OFETs), organic electrochemical transistors (OECTs), and organic photodetectors (OPDs) have presented excellent sensitivity, selectivity, and quick response times, enabling precise detection of target analytes in complex samples. OFET- and OECT-based biosensors are characterized by response times ranging from seconds to a few minutes, which is well suited for monitoring biomolecular interactions in complex biological samples [211].

For example, DNA-based sensing has progressed toward realizing ultrasensitive detection (below 1 × 10^−12^ M or even individual DNA strands), identification of single-nucleotide mismatches for SNP analysis, and effective DNA sequencing. Organic bioelectronic materials have been utilized for highly sensitive, label-free identification of DNA hybridization. For example, Li et al. created a CNT microarray system to identify DNA hybridization occurrences, utilizing multiwalled carbon nanotubes (MWCNTs) cultivated on chromium-patterned electrodes of different sizes (2 µm × 2 µm and 200 µm × 200 µm). Only the CNT tips were uncovered for current conduction, while the remainder were insulated by silicon dioxide. Ferrocene-functionalized single-stranded DNA (ssDNA) probes were immobilized, and hybridization was monitored using cyclic voltammetry (CV), revealing elevated peak voltage resulting from guanine oxidation in the presence of Ru(bpy)_3_^2+^. The density of the CNT array significantly affected the platform’s capability to identify attomolar levels of target DNA [203].

#### 4.1.2. Electrophysiological Recording

The capture of physiological signals is vital for evaluating the health condition not only of patients but also of healthy people, as ongoing monitoring promotes point-of-care diagnostics and preventive healthcare. Main techniques consist of electrocardiography (ECG) for heart issues, surface electromyography (EMG) for muscle function, and electroencephalography (EEG) for brain evaluation. For the achievement of effective signal recording, electrodes must fulfill specific requirements, including provision of good skin contact, non-toxicity, and comfort, and possession of low impedance.

Electrodes based on organic PEDOT:PSS have become a promising substitute for conventional metal electrodes because of their advantageous characteristics, such as low impedance and biocompatibility. PEDOT:PSS-based electrodes typically exhibit lower skin–electrode impedance compared to conventional metal electrodes, resulting in improved signal quality and reduced motion artifacts during ECG and EMG recordings. Recent innovations involve the creation of an affordable, dry PEDOT:PSS-based tattoo electrode that can carry out EMG and ECG recordings on different areas of the body. However, additional enhancements in the biocompatibility of materials and the physical and electronic properties of these electrodes are still required [212].

#### 4.1.3. Cell Interfacing and Tissue Engineering

The relationship between materials and cells plays a pivotal role in applications, such as neuronal activity monitoring, stimulation devices, tissue regeneration scaffolds, drug delivery systems, and cell culture platforms. Consequently, the functionalization of surfaces to enhance these interactions has garnered increasing attention from the researchers in recent years [212].

In bone–tissue engineering, efforts have been made to employ organic conductive substances in osteogenic differentiation. Indeed, improved osteogenic proliferation and differentiation has been documented on graphene, CNTs, and CVD diamond. These conductive carbon-based materials promote enhanced cell adhesion, proliferation, and lineage-specific differentiation by providing electrically active and mechanically supportive microenvironments [175]. Additionally, in another study, GO adorned with extracellular matrix (ECM) proteins, including fibrin, facilitated the differentiation of an osteoblast-like cell line (MG-63) toward mineralization. Moreover, CNTs coated or conjugated with hydroxyapatite, a crucial component in bone formation, have been researched and exhibited encouraging outcomes for potential implantable platforms [203]. Such organic and hybrid biointerfaces are, therefore, considered promising candidates for implantable tissue engineering platforms, where biocompatibility, electrical functionality, and controlled cell–material interactions are critical [184].

### 4.2. Energy Storage and Harvesting

#### 4.2.1. Supercapacitors [47]

Undeniably, the continual growth of biodegradable and recyclable material portfolios for conductive, semiconductive, and dielectric inks and synthetic and natural polymer substrates is promoted by printing and post-processing techniques and optical inspection systems. As a result, printing methods have become popular for large-scale flexible electronics manufacturing, involving radiofrequency identification (RFID) devices, photovoltaic cells, organic light-emitting diodes (OLEDs), thin-film transistors (TFTs), diodes, displays, batteries, and sensors for measuring temperature, humidity, and pH levels. These ongoing advances indicate that credible and reproducible solutions for the large-scale production of PE with well-established fabrication standards will be accessible in the very near future [2]. Printed supercapacitors based on polymeric and carbon-based inks have demonstrated areal capacitances in the range of several mF cm^−2^ and stable charge–discharge behavior over thousands of cycles, supporting their suitability for flexible and disposable energy storage applications [2,45].

#### 4.2.2. Solar Cells

Biopolymers have shown to be sustainable and eco-friendly materials used in the development of photovoltaic devices, especially in dye-sensitized solar cells (DSSCs) and hybrid systems. Chitosan, pectin, cellulose, silk, and DNA can be used as substrates, electrolytes, or structural components, while they provide mechanical strength, low thermal expansion, and excellent transparency at the final product [112,213]. For instance, films made from silk with silver nanowires exhibit excellent optical and electrical properties, better than conventional flexible substrates, such as ITO-PEN. Silk fibroin/silver nanowire substrates exhibit optical transmittance above 85% and sheet resistances comparable to, or lower than, ITO-PEN, while offering superior flexibility and biodegradability [112]. Moreover, electrolytes from starch combined with ionic liquids furnish enhanced ionic conductivity and thermal stability, significantly improving the performance of DSSCs [214,215]. Solid-state DSSCs employing biopolymer-based electrolytes (e.g., starch-, cellulose-, or chitosan-derived systems) have demonstrated power conversion efficiencies in the range of ~3–7%, along with improved thermal stability and reduced leakage compared to conventional liquid electrolytes [185,187,189]. Also suitable for photovoltaic applications are polyvinyl alcohol nanocomposites with SiC nanoparticles, which demonstrate high absorption in the UV and visible spectrum [216]. Overall, biopolymers offer a wide range of functional properties that enable their utilization in efficient, flexible, and environmentally friendly solar cells [217]. Lately, energy devices have been enhanced by the use of biopolymers both as electrodes and electrolytes [102,112]. Biopolymers such as chitosan, cellulose, collagen, and dipeptide nanofibers are used as electrodes because they consist of hydrophilic groups (-OH and -NH_2_), which allow ion adsorption and electric double-layer formation [102,112,213], while treatments such as pyrolysis or embedding nanomaterials (graphene) can lead to increased electrical conductivity and capacitance [112,213].

Except for electrodes, biopolymers are used to develop solid or semi-solid electrolytes, as well. For example, the use of ionic liquids in cellulose- or chitin-based materials has been shown to improve the voltage stability and energy density of SCs, providing stability over the circles of use, at the same time [185]. Finally, the ability to form porous, spongy structures (such as those made of collagen) enables the electrolyte to be encapsulated efficiently, reducing leakage and ensuring long-term performance [213]. Biopolymer-based solid and gel electrolytes have also shown electrochemical stability over hundreds of operating cycles, maintaining stable open-circuit voltage and fill factor, which is critical for the long-term operation of flexible photovoltaic devices [185,190].

#### 4.2.3. Triboelectric Nanogenerators (TENGs)

Triboelectric nanogenerators (TENGs) have the ability to convert mechanical energy into electrical due to the triboelectric effect [186]. Nowadays, biodegradability, flexibility, and environmental compatibility are key characteristics in developing a product so materials such as cellulose, chitosan, silk, and lignin are used, resulting in bio-TENGs [186,187]. Bio-TENGs can be enhanced through surface modifications and incorporation of nanomaterials, approaching the performance of synthetic ones [186]. Their applications include portable medical systems, environmental monitoring, and energy autonomy of portable devices [187,188]. Biobased TENGs fabricated from cellulose, chitosan, silk, or lignin have been reported to generate output voltages of several tens to hundreds of volts and power densities comparable to those of conventional polymer-based TENGs, while offering enhanced biodegradability and environmental compatibility [191,192].

#### 4.2.4. Piezoelectric Devices

Piezoelectric devices have the ability to convert mechanical stress to electrical charges, which makes them very important components in flexible energy systems and portable devices [189]. Polymers such as polylactic acid (PLA) and poly-L-lactide (PLLA) display significant piezoelectric properties, especially when their crystallinity and orientation of the polymer membrane are increased through suitable processing methods, such as high-speed spinning [102]. Especially in applications such as liquid crystal displays and microdevices, PLA’s piezoelectric response is enhanced by ceramic fillers, such as BaTiO_3_ [102]. Other biopolymers, such as PHAs, demonstrate natural piezoelectric activity and have been used in vibration sensors, piezometers, ultrasonic sensors, and energy harvesting devices [190]. However, stability and maximization of piezoelectric properties through nanostructural strategies remain technical challenges [189,191].

### 4.3. Memory and Information Storage

#### Resistive Memory Devices (ReRAM)

Biopolymers, such as chitosan, have shown the ability of developing reproducible and reliable bipolar resistive switching characteristics used in flexible and transparent devices, making them suitable for portable or temporary applications [123]. Biopolymer-based ReRAM devices have demonstrated stable bipolar switching with ON/OFF resistance ratios exceeding 10^2^–10^3^ and data retention times extending beyond 10^4^ s, indicating performance levels suitable for transient and flexible memory applications [197,198,199]. The development of bio-ReRAMs based on natural polymers is contributing to the creation of soluble and physically transient memories with applications in medical implants and green technology [193]. Due to their low power consumption and simple architecture, biopolymer ReRAM devices are considered valuable candidates for the future, sustainable information storage systems [194].

### 4.4. Displays and Photonics

#### 4.4.1. Displays

The emerging need for new sustainable technologies has resulted in wide usage of biopolymers in flexible displays. A typical example is the use of polylactic acid (PLA) reinforced with BaTiO_3_ nanoparticles to fabricate piezoelectric films, which can act as liquid crystal activators, allowing imaging to be achieved without the use of electrodes [102]. Similarly, the injection of semiconducting nanostructures, such as ZnSe:CdTe quantum dots, into biopolymer matrices provides the potential for full-color display capability, enhancing the applicability of biomaterials in modern displays [75].

BC has been exploited as a transparent and flexible substrate for display applications through modification with d-limonene that increases transparency and allows avoiding the use of harmful solvents, while providing the possibility to recover the materials after the disassembly of the device [195]. Similarly, sodium alginate has been used as a flexible substrate, enhancing the circular recovery of materials thanks to the possibility of separating and recycling the individual components [196].

The utilization of biopolymers for transparent, lightweight, and recyclable imaging media promotes sustainability in the electronics industry and makes them attractive as potential materials for applications where eco-performance is as important as functionality. Their ease of molding, their adaptable properties, and the possibility to be combined with nanomaterials enhance their role in the next generation of smart, environmentally friendly imaging devices [197].

#### 4.4.2. Photonic Devices

**Figure 16:**
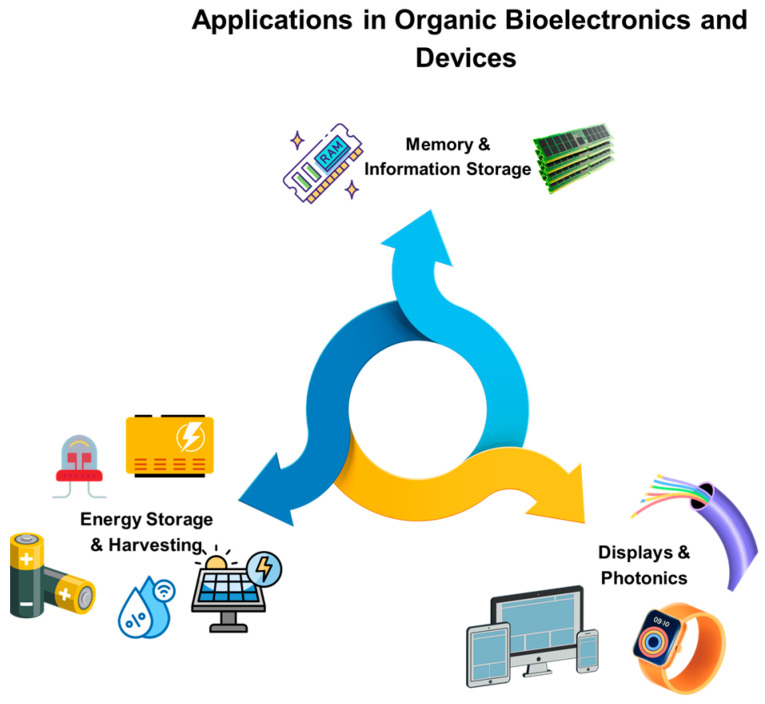
Schematic representation of PE applications in memory and information storage, energy storage harvesting, displays, and photonics.

Biopolymers are promising materials for innovative photonic devices, combining biodegradability, renewability, and multifunctional optical properties. Physical structures, such as chitin from insects and protein fibers, achieve complex light interactions through complex nanostructures that are difficult to reproduce artificially [198]. To approximate such bio-photonic performances, techniques such as biosynthesis and self-assembly of biopolymer are exploited, leading to multilayer, chiral, or opal structures with adjustable optical responses [199]. Furthermore, biopolymers, such as DNA and collagen mixed with photoactive molecules and formed into thin films, are promising for applications in optical switches, photonic memories, and light amplifiers, mainly due to their high photostability [200]. The aforementioned applications are summarized in Figure 16.

## 5. Lifecycle Assessment (LCA)

Lifecycle assessment (LCA) provides a systematic approach for quantifying the cumulative environmental impacts of biobased substrates, and device EoL strategies, enabling direct comparison to fossil-based materials. It highlights the potential of biobased polymers to reduce environmental footprints in PE but also identifies persistent technical, infrastructure, and systemic challenges. Sustainable development of these devices requires combined advances in material science and process engineering, as well as ongoing monitoring of their broader ecological impacts throughout the entire lifecycle.

Recent studies have shown that substituting typical substrates, such as PET or polyimide, with biobased alternatives can reduce the global warming potential (GWP)—the effect of various greenhouse gases into equivalent amounts of carbon dioxide (CO_2_ equation)—of PE, depending on the material and manufacturing choices [206,207].

**Figure 17:**
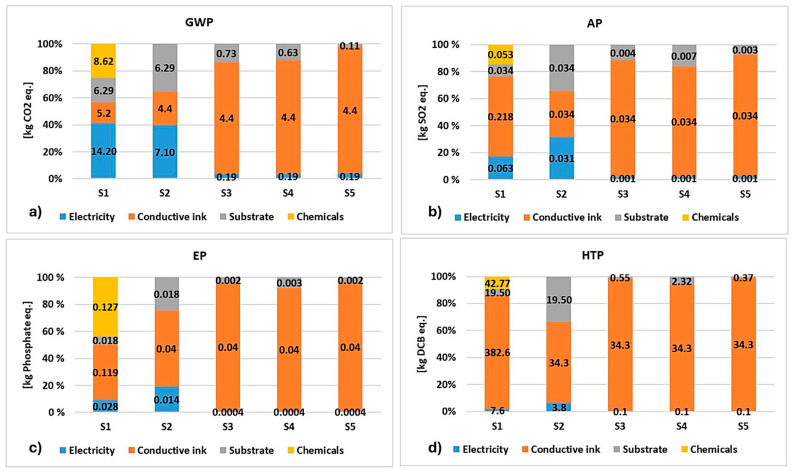
LCA for fiberglass-reinforced brominated epoxy resin (FR4; S1) and a biobased PLA–glass fiber composite (S4) in PCB substrates. The value displayed on each bar represents the corresponding absolute environmental impact. Grey color represents the contribution of the substrates, (**a**) electricity is the main contributor to GWP, at 41%, (**b**–**d**) the conductive material (Ag NPs) is the most significant contributor in all environmental impact categories [208].

Nassajfar et al. [208] investigated, through LCA, the contribution of two different substrates—namely, a fiberglass-reinforced brominated epoxy resin (FR4; S1) and a biobased PLA–glass fiber composite (S4)—to the environmental impacts of PCBs. In the context of GWP, it was demonstrated that the impact is reduced from 6.29 kg CO_2_ in Equation (S1) to 0.63 kg CO_2_ in Equation (S4), respectively (Figure 17a). Similarly, human toxicity potential (HTP) values revealed a dramatic decrease from 19.50 kg DCB in the equation for FR4 to only 2.32 kg DCB in the equation for PLA-GF (Figure 17b), underscoring the lowered toxicological risks to humans associated with biobased substrates. When evaluating the lifecycle of polymers, it is important to recognize that assessing global warming potential alone does not fully capture their environmental impacts. Biopolymers, for example, can have significant contributions to acidification and eutrophication, primarily due to the fertilizers, pesticides, and insecticides used during their agricultural cultivation [209]. In the same work, Nassajfar et al., in the context of acidification potential (AP), observed that the substrate-related impact decreased from 0.034 kg SO_2_ in the equation using FR4 to 0.007 kg SO_2_ in the equation with the PLA-GF substrate (Figure 17c). Similarly, for the eutrophication potential (EP) indicator, the substrate’s contribution declined from 0.018 kg in the phosphate equation to 0.003 kg in the phosphate equation for the same case (Figure 17d). This demonstrates that adopting biobased substrates also yields substantial reductions in both acidification and eutrophication burdens, offering a thorough assessment of material sustainability in electronic applications [208].

In another study, Zikulnig et al. conducted an LCA on various biobased substrates for printed sensor tags. Surprisingly enough, the results showed that biobased polyethylene (bio-PE) demonstrated the highest GWP among these substrates, at −3.53 kg CO_2_ eq/kg, compared to −1.33 kg CO_2_ eq/kg of fully biobased PET and 1.06 CO_2_ eq/kg of PLA, thus making it potentially the most climate-friendly option in this set [210]. These results contradict with the study of Shen and his group, who reported GWP of 1.36 kg CO_2_ in the equation per kg for bio-PET and 1.30 kg CO_2_ in the equation per kg for PLA [211].

End-of-life management is also pivotal for minimizing the lifecycle impact. Recycling presents the best environmental outcome but is limited by available infrastructure and the complexity of material recovery when multiple layers and components are integrated. Välimäki and her team demonstrated a lower carbon footprint of recycled PET (rPET) against PLA as substrates in ultra-thin organic photovoltaics (OPV), suggesting that rPET has a decreased climate impact [6].

## 6. Challenges and Future Outlook

Biobased materials can have a major role in the upcoming green transition of the electronics industry owing to their huge potential for sustainable upgrading [3]. However, multifaceted and significant challenges in utilizing biobased polymers as substrates for industrial-scale PE applications exist, despite the growing interest in sustainable materials [210].

Today, the production of conventional plastics is highly advanced, operates on a much larger scale, and remains significantly more cost-effective than biobased polymers [212]. Technically, a primary challenge lies in developing biobased substrates that possess superior dimensional and heat stability and solvent and mechanical performance. Bio-derived polymers, especially proteins and certain polysaccharides, are typically highly hydrophilic, making them susceptible to swelling or structural degradation caused by moisture under changing hygroscopic conditions. PE often subject substrates to high temperatures and exposure to solvents or chemicals—conditions under which conventional substrates like PET perform excellently [52]. A practical research pathway to address limitations in mechanical strength is the adaptation of polymer-processing strategies demonstrated by Luomo et al., including biaxial orientation and thermal annealing, which enable bio-PET and PLA to reach mechanical strength levels comparable to commercial PET [52]. Additionally, the mechanical properties and heat resistance of PLA can be enhanced through approaches such as nucleation, stereochemical modification, or the incorporation of additives [2]. In parallel, low-temperature sintering methods, such as intense pulsed light sintering, enable conductive patterning on heat-sensitive biobased substrates while increasing the electrical conductivity [2].

To address the inherent challenge of long-term stability in wet environments, specific stabilization strategies must be incorporated. Chemical cross-linking (e.g., using glutaraldehyde for chitosan) and the application of functional coatings, such as heat-sealable layers on regenerated cellulose, provide the necessary moisture barriers. Furthermore, as explored in the context of transitional electronics, the use of encapsulation strategies where the thickness of the protective layer determines the lifetime of the device allows for strategic management of device durability before degradation begins.

Economic viability remains a major obstacle to the broad adoption of biobased polymers in PE, as their costs are generally higher than those of petrochemical-based materials. Bioplastics face challenges in formulation, processing, and end-of-life management, which hinder their ability to compete with low-cost conventional plastics [213]. Even though some affordable biobased polymers exist, they have yet to fully replace petroleum-derived plastics due to financial constraints affecting both manufacturers and consumers [45]. For example, the development of green technologies for chitosan- and cellulose-derived products is constrained by both the limited availability of these raw materials and the typically environmentally burdensome nature of their manufacturing processes. Importantly, the choice of source for cellulose and chitosan directly influences the final cost of the bioplastics. Integrating these materials into existing production lines may require significant equipment adaptation and process revalidation, leading to increased capital and operational expenditures [214]. Additionally, the manufacturing of bioplastics is often less energy-efficient compared to traditional plastic production methods, adding to cost and sustainability concerns [11]. Achieving a balance requires increasing production to lower costs and implementing regulatory frameworks, such as carbon taxes or mandatory e-waste recycling fees, which compel companies and consumers to account for the climate-related costs of petrochemical materials, thereby fostering greater adoption of sustainable alternatives [215].

Sourced from renewable resources, biopolymer materials show sustainability benefits compared to petrochemical polymers: unlimited availability, non-toxicity, and biodegradability [201]. For this reason, it is only fair to assess the benefits and drawbacks of the biopolymers compared with non-biopolymers in the field of printed electronics. In the following paragraphs the limitations of biobased polymers in printed electronics are also discussed. In terms of electrical properties, biopolymer materials, such as cellulose, PLA, silk fibroin, and PCL, have been applied due to requirements of biodegradability, biocompatibility, or sustained biodegradation [201,202]. For instance, PLA was used for the development of functional thin-film transistors’ circuits [201]. Silk fibroin composites have also produced biodegradable TCEs. Additionally, PCL substrates have also been used for biodegradable strain and acoustic sensors [201]. In addition, biobased substrates, such as PLA and cellulose acetate propionate, have demonstrated printability and electrical performance comparable to PET when processed under optimized, lower-temperature curing conditions [113,202].

In terms of electrical performance, biobased materials for printed electronics tend to be limited compared to traditional polymers since they naturally possess low conductivity with unharvested charge transport channels [203]. Using biopolymer-based binders in the formulation of conductive inks tends to add to the resistivity, sometimes making post-printing treatments necessary to attain adequate conductivity [12].

Among the weaknesses of biobased materials for printed electronics compared to conventional petroleum-based polymers, one can find the requirement for improvement concerning the formulation of inks. Biobased inks tend to be thicker, drier, and harder to process, showing low dispersibility, thus potentially undermining printing quality and the scalability of manufacturing. Compatibility with conventional roll-to-roll processing equipment in manufacturing lines is also less well-developed than that of more conventional, well-established polymer materials [12]. The major shortcomings of biobased materials within printed electronics include low stability, low electrical performance ceiling, and high costs [201,203]. Enhanced degradability of biobased polymers, although being a major advantage concerning the end-of-life treatment of printed devices, tends to undermine the stability of printed devices upon exposure to the ambient conditions of temperature, water, or sunlight stress [12,202]. Furthermore, many biopolymers have lower thermal resistance, restricting post-printing annealing, soldering, and device integration strategies [113]. Cost and scalability also remain challenges, as several biobased substrates and specialty polymers are not yet produced at the scale or price of conventional plastics.

Biodegradability is inherently connected to chemical and physical susceptibilities—like hydrolysis, oxidation, enzymatic activity, and ultraviolet exposure—that can reduce the long-term mechanical and electrical properties required for reliable electronic operation [150,204]. Consequently, the same processes that enable eco-friendly degradation post-end-of-life can initiate early decline in device performance while in use, especially in humid, wet, or biologically active conditions.

As far as natural polymers are concerned, they usually present limited mechanical strength and susceptibility to moisture, whereas synthetic biodegradable polymers offer enhanced adjustability in flexibility, stretchability, and conformal adhesion with dynamic surfaces like biological tissues [201,204]. Nevertheless, the achievement of a controlled degradation process—such as conjugation-breaking reactions—while maintaining stable electrical properties over the desired operational lifetime is still a major challenge, even in synthetic conjugated polymers used as electrically active layers [204]. Actually, contact with biofluids or moisture diminishes the electrical performance, and this process precedes observable mass loss, accentuating the sensitivity of electronic functionality to initial degradation processes [150].

To reduce premature failure, focus has been directed toward encapsulation techniques and kinetic regulation of material dissolution [201,203]. Biodegradable encapsulation layers, consisting of organic and inorganic coatings, are often used to facilitate delayed water penetration and maintenance of the device’s performance. However, these approaches present additional trade-offs: inorganic barriers deliver enhanced moisture diffusion resistance but are prone to brittleness, whereas organic encapsulants ensure mechanical flexibility but compromise on water-blocking effectiveness [203].

Lastly, the end-of-life management and environmental impact of biobased substrates need further clarification. Biodegradability influences the environmental impacts at the end-of-life stage and affects how materials are disposed of. Nevertheless, it is important to note that not all biobased polymers are biodegradable, recyclable, or compostable. The distinction between biobased origin and biodegradation capability means that while some biobased polymers can break down naturally, others may require alternative management strategies to minimize the environmental footprint [216]. PLA, a widely used bioplastic, requires industrial composting conditions with higher temperatures than those found in natural environments, meaning it does not effectively biodegrade in home compost bins. Its industrial composting infrastructure is limited and its degradation behavior within electronic devices requires thorough assessment to ensure safety and performance over the product lifecycle. This limitation restricts PLA’s market growth and effectiveness, suggesting that without addressing these challenges, it may be phased out. Overall, a lack of awareness about appropriate waste management of biobased polymers limits their environmental benefits [217].

## 7. Conclusions

The current review paper underscores the vital role of biobased polymers in advancing sustainable PE, driven by increasing research attention to reduce reliance on fossil resources, electronic waste, and environmental impact.

Among the most studied materials are chitosan, PLA, PHAs, silk, starch, cellulose, and their derivatives. Given that biobased polymers often exhibit inferior properties in their pure form or in blends compared to conventional materials, incorporating fillers and nanofillers to produce composites has become a key strategy, enhancing mechanical strength, conductivity, and thermal stability for PE applications.

Looking ahead, the development of biobased polymers for printed electronic substrates holds great promise, aligned with sustainability objectives. Nevertheless, substantial improvements are still needed before commercial-scale manufacturing becomes feasible, as challenges related to stability, reproducibility, and large-scale performance persist. Advances in formulation and processing techniques are expected to improve the mechanical, thermal, and chemical robustness of these materials, supporting their integration into industrial printing methods, such as inkjet and screen printing. Additionally, employing green synthesis and modification approaches could enhance economic viability by lowering costs and environmental impacts.

In summary, the renewable origin of biobased polymers makes them especially attractive in the context of escalating environmental concerns and the global transition toward sustainable technologies. Continued innovation and development of these materials represent a crucial step toward realizing greener and more sustainable printed electronic systems.

## Figures and Tables

**Table 1:** Summary of biobased polymer blends and their electronic applications.

Application	Blend Type	Key Findings	Performance Data	Reference(s)
Flexible substrates for electronic and gas-sensing devices	Chitosan/Poly(vinylpyrrolidone) (PVP)	Flexible substrates for gas-sensing and semiconducting devices	Tensile strength of 57.8 N/mm^2^, transmittance (550 nm) > 90%, comparable to commercial PET flexible substrates	[91,100]
Flexible and wearable optoelectronic devices	Chitosan/Potato starch	Substrates for flexible and wearable electronics	Maximum tensile strength at 30.2 MPa and elongation at 35.6%, both higher than pure starch and chitosan films	[86,101]
Conductive materials/electroactive composites	Carboxymethyl cellulose (CMC)/Polyaniline	Enhanced electrical conductivity observed in blends	Higher thermal stability of blends (TGA analysis): 40–43%, final remaining mass vs. 22% for polyaniline and 35% for CMC, higher electrical conductivity (S/cm) with CMC increase: (503 K) CMC30% → 0.442 < CMC40% → 0.934 < CMC50% → 2.32	[89,90,100]
Conductive substrates	Bacterial cellulose/Polyaniline	Improved electrical conductivity similar to CMC/polyaniline systems	Blends with drained bacterial cellulose: ~140 × 10^−3^ S/cm	[102]
PE (general)	Poly(lactic acid) (PLA)/Poly(hydroxybutyrate) (PHB)	Tested for inkjet and screen printing; doped with lignin and CNTs for EMI shielding (18–26.5 GHz)	More cycles of dip coating in CNT suspension ⇒ lower sheet resistance (one step: ~10 kΩ/□ > six steps: ~1 kΩ/□); therefore, higher conductivity	[94]
Dielectric layers for OTFT and MIM capacitors	Poly(vinyl alcohol) (PVA)/Carrageenan (CAR) and PVA/Poly(ε-caprolactone) (PCL)	Bilayer dielectric stacks; enhanced mechanical integrity and dielectric properties	PVA/CAR: dielectric constant of 26, ON/OFF ratios of 104–105, low leakage currents (38 nA), PVA/PCL: dielectric constant (2) and contact angle (128°) comparable to conventional materials (e.g., PTFE ~ 2 and 121°)	[95]

**Table 2:** Summary of cellulose-based substrates and their applications.

Cellulose Substrates	Application	Key Findings	References
Nanocellulose papers (NCPs)	Green PE (inkjet printing, screen printing, and direct ink writing)	Low sheet resistance, good flexibility in bending tests, but insufficient surface flatness	[97]
Ethyl cellulose/hydroxypropyl cellulose blend	Flexible electronic substrates (UV photolithography and screen-printing potential)	Environmentally friendly production, tunable mechanical/chemical properties, excellent stability and elongation	[3]
Methylcellulose + tannic acid hydroplastic	Flexible printed circuit boards (FPCBs) with 3D-printed sensors	Recyclable, soluble in medical alcohol, thermally and mechanically stable even underwater, durable sensing performance	[116]
Wood-based cellulose with preserved nanofiber alignment	Flexible printed circuits (lignin-derived carbon nanofibers as conductive ink via standard printer)	Flexible, high tensile strength, transparent, conductive, suitable for sensors and wearables	[117]

**Table 3:** Quantitative comparison between fossil-based and lignin-based graphene and CNTs [144,145,146,147,148,149,150,151].

Properties	Fossil-Based Graphene	Fossil-Based CNTs	Lignin-Based Graphene	Lignin-Based CNTs
Electrical conductivity (S/m)	10^7^–10^8^	10^6^–10^8^	10^1^–10^4^	1.19 × 10^5^
Tensile strength (GPa)	130	63–100 (tubes) 1 (fibers)	0.35–1.7	0.4–1.3
Thermal conductivity (W/mK)	5000	3000–5000	0.28	33

**Table 4:** Biobased additives and composites with applications in PE.

Composite/Material	Properties	Applications	Reference
PHBV/wood-derived carbon fillers	Enhanced electrical conductivity	Electronic devices	[174]
PLA/biochar	Desirable electrical conductivity, decreased mechanical properties	PE	[175]
Ethyl cellulose/biochar	High roughness and porosity, mechanical stability, desirable rheological properties	Screen-printed electrodes	[176]
Lignin-derived CNTs/Cu yarns	Enhanced electrical conductivity, mechanical properties	Wearable and flexible electronics	[177]
Bio-graphite derived from coconut coir	High graphitization	Lithium-ion batteries	[178]
Eucalyptus oil and ferrocene derived CNTs	High thermal stability, low surface area	Electronic devices	[131]
Bio-graphite from char/graphene nanoplatelets	High crystallinity, energy efficiency	Screen-printable graphene inks	[179]
TPU/ball-milled biochar	High mechanical and thermal properties	Electronic devices	[180]
Starch-derived hard carbon microspheres	Enlarged interlayer spacing, reversible capacity, coulombic efficiency	Na-ion batteries	[158]

**Table 5:** Comparative overview of key physical, electrical, and processing properties of biobased polymers for printed electronics [184,185,186,187,188,189].

Material/Blend	Role in PE	Dielectric and Electrical Properties	Optical Transparency	Stability (Thermal/ Environmental)	Processing Window/Techniques	Degradability/EoL Impact
PLA	Substrate/3D printing filament	Low Є (~2.5–3.1)	High (up to 94%)	Tm: 150–176 °C; stable in UV-A and thermal cycles	FDM, screen, R2R; drying at 80–100 °C	GWP: 0.63 kg CO_2_ eq (vs 6.29 for FR4); industrial composting
Cellulose	Flexible substrate	High Є (~3.9–7.5)	>80%	High thermal resistance (Tm: 200–250 °C); stable under UV-A and humidity	Inkjet, screen, DIW; compatible with thermal annealing and silver ink sintering	Soil and marine degradation; facilitates component recovery
Alginate	Sustainable electrolyte/Gel	Proton conductivity: ~5.5 × 10^−3^ S/cm; sustainable electrolyte; capacitance: ~2.0 μF/cm^2^	~92%	Hydrogel formation; sensitive to humidity/moisture (high water absorption)	Stencil-printed liquid metal; soft electronics applications	Biodegradable hydrogel; non-toxic disposal
Chitosan	Substrate/Shielding layer	High EMI shielding: 25–60 dB; dielectric substrate capability	Transparent films	High thermal stability (up to ~200 °C); moisture barrier properties	Solution-processable bio-inks	Fully biodegradable, non-toxic
Lignin (Carbonized)	Conductive filler/Precursor	High conductivity (~10^3^–10^8^ S/m)	Opaque (carbonaceous)	Decomposition at 200–500 °C	Carbonization via pyrolysis or laser treatment	Valorization of biomass waste; circular solution in PE

## Data Availability

No data were used for the research described in the article.

## Abbreviations

The following abbreviations are used in this manuscript:

Full Name	Acronym
Acrylonitrile Butadiene Styrene	ABS
(3R,6S)-hexahydrofuro[3,2-b]furan-3,6-diyl bis(1,3-dioxo-1,3-dihydroisobenzofuran-5-carboxylate)	ISBESA
3D-Printed Nanocomposite Graphene Electrodes	3D-nGEs
Bacterial Cellulose	BC
Carbon Fiber Reinforced Polymer	CFRP
Carbon Nanotubes	CNTs
Carboxymethyl Cellulose	CMC
Carrageenan	CAR
Cellulose Acetate	CA
Cellulose Nanocrystals	CNCs
Cellulose Nanofibrils	CNFs
Chitosan-Poly(vinylphosphonic acid)	PVPA
Chitosan/Graphene/Gold Nanoparticles	AuNP
Poly(vinylpyrrolidone)	PVP
Cold Spray	CS
Compound Annual Growth Rate	CAGR
Continuous Inkjet Printing	CIJ
Direct Ink Writing	DIW
Drop-on-demand	DOD
Dye-Sensitized Solar Cells	DSSCs
Electrocardiography	ECG
Electroencephalography	EEG
Electrostatic Assist System	ESA
End-of-life	EoL
Ethyl Cellulose	EC
European Union	EU
Fiberglass-Reinforced Brominated Epoxy Resin	FR4
Field-Effect Transistors	FEs
Flexible Organic Field-Effect Transistors	OFETs
Flexible Printed Circuit Boards	FPCBs
Flexographic Printing	FP
Fused Deposition Modeling	FDM
Global Warming Potential	GWP
Graphene Oxide Nanoparticles	GO NPs
Gravure Printing	GP
High-Heat PLA	hhPLA
Hydroxypropyl Cellulose	HPC
Lifecycle Assessment	LCA
Light-Emitting Diodes	LEDs
Liquid Crystal Display	LCD
Metal-Insulator-Metal	MIM
Microcrystalline Cellulose	MCC
Nanocellulose Papers	NCPs
Nanofiber Cellulose	NFC
Organic Electrochemical Transistors	OECTs
Organic Field-Effect Transistors	OFETs
Organic Light-Emitting Diodes	OLEDs
Organic Photodetectors	OPDs
Organic Photovoltaics	OPVs
Organic Thin-Film Transistors	OTFT
Photovoltaic Cells	PV Cells
Poly(3,4-ethylenedioxythiophene)	PEDOT
Poly(3,4-ethylenedioxythiophene) Polysulfonate	PEDOT:PSS
Poly(3-hexythiophene)	P3HT
Poly(hydroxyalkanoate)	PHA
Poly(3-hydroxybutyrate)	PHB
Poly(3-hydroxybutyrate-co-3-hydroxyvalerate)	PHBV
Poly(dimethylsiloxane)	PDMS
Poly(ether imides)	PEI
Poly(ether sulfones)	PES
Poly(ether-ether-ketone)	PEEK
Poly(ethylene naphthalate)	PEN
Poly(ethylene terephthalate)	PET
Poly(p-phenylene vinylene)	PPV
Poly(vinyl alcohol)	PVA
Poly(vinylidene fluoride)	PVDF
Poly(ε-caprolactone)	PCL
Polyaniline	PANI
Polyhydroxyalkanoates	PHAs
Polyimides	PI
Polylactic Acid	PLA
Polylactic-Co-Glycolic Acid	PLGA
Poly-L-lactide	PLLA
Polypropylene	PP
Polytetrafluoroethylene	(PTFE)
Polypyrrole	PPy
Polysulfonates	PSS
Polyurethane	PU
Printed Circuit Boards	PCBs
Polyethylene	PE
Proton Exchange Membranes	PEM
Radiofrequency Identification	RFID
Resistive Memory Devices	ReRAM
Reverse Offset Printing	ROP
Roll-to-Roll	R2R
Roll-to-Sheet	R2S
Sheet-to-Sheet	S2S
Silk Fibroin	SF
Spray Coating	SC
Stereocomplex PLA Blends	scPLA
Surface Electromyography	EMG
Thermoplastic Polyurethanes	TPU
Thin-Film Transistors	TFTs
Transparent Wood Film	TWF
Triboelectric Nanogenerators	TENGs
Ultrasonic Nozzle Spraying	US
Ultraviolet	UV
United Nations	UN
